# Quantitative Susceptibility Mapping of Deep Grey Matter in MS: Association With Clinical Scores and Brain Volume Measures

**DOI:** 10.1002/brb3.70988

**Published:** 2025-10-20

**Authors:** Cui Ci Voon, Jakob Meineke, Tun Wiltgen, Julian McGinnis, Ronja Berg, Christine Preibisch, Sarah Schlaeger, Benedikt Wiestler, Christina Engl, Achim Berthele, Jan S. Kirschke, Bernhard Hemmer, Mark Mühlau

**Affiliations:** ^1^ Dept. of Neurology, School of Medicine and Health Technical University of Munich Munich Germany; ^2^ TUM‐Neuroimaging Center, School of Medicine and Health Technical University of Munich Munich Germany; ^3^ Philips Innovative Technologies Hamburg Germany; ^4^ Dept. of Neuroradiology, School of Medicine and Health Technical University of Munich Munich Germany; ^5^ AI For Image‐Guided Diagnosis and Therapy, School of Medicine and Health Technical University of Munich Munich Germany

**Keywords:** atrophy, clinical severity, DGM, iron deposition, multiple sclerosis, QSM

## Abstract

**Background:**

Changes in quantitative susceptibility mapping (QSM) of the deep grey matter (DGM) in multiple sclerosis (MS) are thought to reflect tissue damage invisible in conventional magnetic resonance imaging (MRI) sequences, such as iron‐related neurodegeneration.

**Objective:**

To explore the associations of clinical scores and MRI‐based volumes with QSM values in the DGM (thalamus, putamen, caudate, and pallidum) in a large cohort of people with MS, and to assess the predictive value of QSM values for clinical outcomes after three years.

**Methods:**

A total of 771 MS patients (clinically isolated syndrome (CIS): *n* = 35, relapsing‐remitting: *n* = 637, progressive: *n* = 63) were scanned at 3T with T1‐weighted, T2‐FLAIR, and QSM sequences. All patients were included in the cross‐sectional analyses examining the relationship of DGM QSM values with MRI variables and clinical scores. Normalized brain volume (NBV) was computed using SIENAX, while total lesion volume (TLV) was derived from the lesion segmentation tool, LST‐AI. Clinical scores included the expanded disability status scale (EDSS), timed 25 foot walk (T25FW), Nine Hole Peg Test (9HPT), symbol digit modalities test (SDMT), and fatigue scale for motor and cognitive functions (FSMC). In the longitudinal analyses, only clinical scores were included, with various sample sizes across different clinical scores (*N*_EDSS_ = 396, *N*_T25Fw_ = 284, *N*_9HPT_ = 284, *N*_SDMT_ = 165, *N*_FSMC_ = 288). These analyses evaluated the predictive value of baseline DGM QSM values for follow‐up clinical scores; these associations were compared to those of corresponding regional DGM volumes.

**Results:**

At baseline, after adjusting for confounding factors, higher QSM values in the basal ganglia were significantly associated with greater TLV (β = 0.14 – 0.17; *p* < 0.001), higher clinical severity (EDSS: β = 0.13 – 0.19, *p* < 0.001), worse dexterity (NHPT: β = 0.14 – 0.16, *p* = 0.02), and lower cognitive functioning (SDMT: β = –0.13 – –0.15, *p* = 0.03). In contrast, lower thalamic QSM values were associated with greater TLV (β = –0.07; *p* = 0.03). Unlike regional volumes, DGM QSM values did not predict clinical outcomes at follow‐up.

**Conclusion:**

DGM QSM values are robustly associated with MS severity and TLV cross‐sectionally. However, our large‐scale longitudinal analysis suggests that DGM QSM values lack prognostic value for short‐term clinical progression in early‐stage MS.

## Graphical abstract

At the cross‐sectional level, quantitative susceptibility mapping detects multiple sclerosis‐tissue damage and clinical severity. However, in contrast to regional deep grey matter volumes, quantitative susceptibility mapping does not predict clinical decline in early MS within a median 3‐year follow‐up.

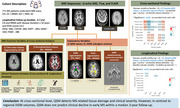

## 1 Introduction

QSM is an advanced MRI technique that quantifies tissue magnetic susceptibility, reflecting the underlying composition of paramagnetic iron and diamagnetic myelin content in the brain. Hence, this method enables the in vivo study of iron deposition in DGM in multiple sclerosis (MS), which is linked to neuroinflammation and neurodegeneration (Haider et al. 2014). Histopathological studies have demonstrated increased iron content in the DGM in MS, correlating with increased susceptibility values (QSM values) (Sun et al. 2015). This supports the use of QSM as an in vivo, non‐invasive method to quantify iron content.

In a recent systematic review (De Lury et al. 2023) and our recent meta‐analysis (Voon et al. 2024), studies have consistently shown significant QSM value changes in MS compared to healthy controls (HC). Specifically, elevated QSM values have been observed in the basal ganglia (caudate, putamen, and globus pallidus), suggesting iron accumulation likely due to disrupted iron homeostasis and oxidative injuries in these regions (Haider et al. 2014, Wisnieff et al. 2015). With the basal ganglia structures consisting mainly of subcortical nuclei and containing little myelin, QSM changes are regarded as an indicator of iron‐driven pathology. Further research also proposed increased QSM values in the basal ganglia may be dependent on regional atrophy, resulting in higher iron concentration instead of the actual increase in iron content (Schweser et al. 2018, Pontillo et al. 2021).

Conversely, decreased thalamic QSM values have been noted in MS patients compared to HC (Voon et al. 2024). This may be due to its overall higher myelin content, which decreases in MS, and the MS‐related depletion of iron stored within oligodendrocytes following oxidative damage. The thalamus' extensive connectivity throughout the brain also makes it particularly vulnerable to secondary neurodegeneration, including Wallerian degeneration (Kipp et al. 2015). A study combining diffusion MRI and QSM showed that thalamic atrophy in MS is associated with widespread microstructural damage, whereas other DGM regions, like the putamen and caudate, did not exhibit significant atrophy (Krijnen et al. 2023). Additionally, Hidalgo de la Cruz et al. (2021) reported that cortico‐thalamic disconnections in patients with CIS predominantly occurred near the third ventricle, indicating early vulnerability of the thalamus to cerebrospinal fluid‐mediated inflammatory processes. This aligns with histopathological findings of a sub‐ependymal gradient of neuro‐axonal loss and microglial activation in the thalamus of progressive MS, further implicating chronic, compartmentalized inflammation as a key pathological mechanism in MS (Hametner et al. 2013). These findings highlight the distinct pathological trajectories in the basal ganglia and thalamus in MS, which are observable even in the early stages of the disease, including CIS (Hidalgo De et al. 2021, Langkammer et al. 2013) and pediatric MS (Mesaros et al. 2008, De Meo et al. 2021), and which can be effectively detected using QSM imaging.

Despite the cumulative evidence on abnormal iron concentrations reported in DGM in MS compared to HC (Voon et al. 2024) and neuromyelitis optica spectrum disorder (NMOSD) (Yan et al. 2021), the relevance of DGM QSM values in relation to MS clinical severity and MRI parameters remains unclear. Only a few studies reported the association of QSM values with MRI parameters, namely with lesion volume (Burgetova et al. 2017, Chiang et al. 2018) and regional DGM volume (Pontillo et al. 2019, Zivadinov et al. 2018). Moreover, findings on the relationship between QSM values and the Expanded Disability Status Scale (EDSS) were inconsistent. While some studies found a positive correlation between QSM values in the basal ganglia and EDSS scores (Burgetova et al. 2017), others did not (Hagemeier et al. 2018). These discrepancies may arise from smaller sample sizes or the lack of control for confounding factors such as age and disease duration. Of note, robust evidence has reported increased iron content and DGM QSM values with age in healthy participants (Hallgren and Sourander 1958, Madden and Merenstein 2023), highlighting the need to control for age.

This study aimed to investigate how QSM values in the DGM (thalamus, caudate, putamen, and pallidum) correlate with clinical scores (physical, cognitive functioning, and fatigue) and MRI surrogates (brain volume and white matter lesion volume) in MS, while adjusting for age, sex, regional DGM volumes, and disease duration. We also investigated whether baseline DGM QSM values predicted MS clinical outcomes at follow‐up. Moreover, we used regional DGM volumes as a reference to evaluate the explanatory strength of QSM values.

## 2 Methods

### 2.1 Participants

This retrospective study, approved by the local ethics committee and adhering to the Declaration of Helsinki, included patients meeting the following criteria: (1) written informed consent for the scientific use of data obtained in clinical routine; (2) diagnosis of MS according to 2017 McDonald criteria (Thompson et al. 2018) or CIS; (3) absence of medical conditions associated with brain pathology, e.g., stroke or brain tumor; (4) no steroid use within 30 days prior to MRI; (5) artifact‐free acquisition of the three MRI sequences used (routinely acquired at our institution between 2019 and 2021); and (6) at least one clinical score within two months before or after baseline MRI scan. For longitudinal analyses, clinical scores had to be obtained at least one year after the baseline scores. If more than one follow‐up investigation was available, the latest was chosen. To reduce baseline variability and to ensure that QSM prediction of the clinical course would be meaningful, we focused on the early phase of MS. Accordingly, we included patients with a disease duration of up to ten years (Leray et al. 2010) but a still unrestricted walking distance (EDSS score of 3.5 or lower) (Noseworthy and Kirkpatrick 2005); further, we only analyzed the CIS/RRMS group as a whole because of the small sample sizes of the subgroups of SPMS and PPMS.

### 2.2 MRI Protocol and Image Processing

All MRI scans were obtained at a Philips Ingenia 3T scanner (Philips Healthcare, R5.6.1.0, Best, NL) with a 32‐channel head coil. The protocol consisted of three main sequences: (1) 3D sagittal T1‐weighted (T1w) magnetization prepared rapid acquisition of gradient echo (MPRAGE): repetition time (TR) = 9 ms, echo time (TE) = 4 ms, flip angle (FA) = 8°, voxel size = 0.75 × 0.75 × 0.75 mm^3^; (2) sagittal 3D fluid‐attenuated inversion recovery (FLAIR): TR = 4800 ms, TE = 317 ms, TI = 1650 ms, FA = 90°, voxel size = 0.75 × 0.75 × 0.75 mm^3^; and (3) For QSM, axial 3D multi‐echo GRE sequence: TR = 41.75 ms, first TE = 6 ms, echo spacing = 6.4 ms, number of echoes = 6 monopolar echoes, flip angle = 20°, FOV = 230 × 187 × 170, acquired matrix = 328 × 267, acquisition voxel size = 0.7 × 0.7 × 1.5 mm, reconstructed voxel = 0.5 × 0.5 × 0.75 mm, pixel bandwidth = 217 Hz/pixel, flow compensation = first echo, CS‐SENSE = 8, elliptical *k*‐space shutter = yes, scan duration = 3 min.

#### 2.2.1 QSM Reconstruction Using Multi‐Echo Complex Total‐Field Inversion

We employed a new approach, multi‐echo complex total field inversion (mTFI), to reconstruct QSM images directly from multi‐echo complex data (see Boehm et al. (2022) and Wen et al. (2021) for related approaches). This method uses total variation regularization with weights derived from the gradient of the echo‐time weighted image magnitudes, similar to MEDI (Liu et al. 2012). The non‐convex optimization process is initialized using a total field inversion (Liu et al. 2017). mTFI offers more robust QSM images compared to two established QSM pipelines, MEDI and STI‐iLSQR (Li et al. 2015), particularly in reducing noise and improving background field removal. Further details on the inverse problem equation for mTFI and the evaluation and comparison of processing pipelines are provided in Supplementary Information Sections 1, 2 and Supplementary Figures 1–4.

#### 2.2.2 Brain and Lesion Volume

Total brain volume, represented by NBV, was computed from baseline native T1w images using the SIENAX toolbox from the FMRIB software library (FSL) (Smith et al. 2002), while FIRST toolbox was used to compute regional DGM volumes (Patenaude et al. 2011). The total intracranial volume (TIV) was determined through the reverse MNI brain mask method (Keihaninejad et al. 2010). TIV was used to adjust DGM volumes for head size through division by the individual TIV and, to remain an intuitive scale, multiplication by the mean TIV of the cohort under investigation. T1w images were first registered to QSM space using FSL FLIRT, then FSL FIRST was employed to process the registered T1w images to generate regional DGM masks for mean QSM value extraction. The cerebrospinal fluid mask, obtained from SIENAX, was used to extract QSM reference values (Smith et al. 2002). Total white matter lesion volume (TLV) was segmented using the lesion segmentation toolbox‐artificial intelligence (https://github.com/CompImg/LST‐AI), based on FLAIR and T1w images, with a minimum lesion volume threshold of 15 mm^3^ (Wiltgen et al. 2024), corresponding to a diameter of 3 mm (Thompson et al. 2018).

### 2.3 Patient Characteristics, Clinical, and MRI Variables

Patient demographics (age and sex), MS disease characteristics (disease duration, MS subtype, clinical scores), and treatment information were collected from routine clinical visits. Baseline explanatory variables included regional DGM QSM values or regional volumes. MRI outcome variables (MRI surrogates) included NBV and TLV. To achieve normal distribution, TLV was logarithmically transformed.

Clinical outcome variables in both baseline and follow‐up analyses included the EDSS (Kurtzke 1983), Nine‐Hole Peg Test (NHPT) (Mathiowetz et al. 1985), T25FW (Motl et al. 2017), SDMT (Smith 1982), and the total score of the fatigue scale for motor and cognitive functions (FSMC) (Penner et al. 2009).

### 2.4 Statistical Analysis

Our main analyses were three types of correlation analyses. First, we performed simple correlations across all variables, using Spearman's rank correlation, accounting for non‐normally distributed variables. Second, to evaluate the relation of DGM QSM values with clinical scores and MRI surrogates, adjusting for age, sex, and disease duration, we used multiple linear regression models. To provide a frame of reference for the effect sizes of QSM values and to account for the confounding influence of DGM volumes, which are related to MS disability (Eshaghi et al. 2018), we analyzed the analogous effect sizes of regional DGM volumes. For each DGM structure, we separately assessed three regression models, using (1) QSM values only; (2) regional DGM volumes only; and (3) both QSM values and regional volumes as explanatory variables. To ensure all variables were suitable for model inclusion, we assessed potential collinearity using the variance inflation factor (VIF). All variables had VIF values below 3, well below the critical threshold of 10 (O'brien 2007). Other regression assumptions were evaluated by visually inspecting residual plots for homoscedasticity and linearity. Potential deviations from normality were addressed using permutation testing with 10,000 iterations. No major violations of linearity or homoscedasticity were observed. Since effect sizes did not considerably differ across models (model 1 vs. 3, and model 2 vs. 3) and as the third model yielded independent contributions, we only report the third here. These cross‐sectional models were:

Outcome variable at baseline = β_o_ + β_1_ (baseline QSM values) + β_2_ (baseline regional volumes) + β_3_ (baseline age) + β_4_ (sex) + β_5_ (baseline disease duration) + *ϵ*

Third, to evaluate the predictive value of DGM QSM values on clinical outcomes, multiple linear regression was employed to compute the associations of baseline QSM values and regional volumes with follow‐up clinical measures, adjusting for age, sex, disease duration at baseline, and follow‐up intervals. Note that to account for disease stage, baseline outcome variables were also included in these longitudinal models. Again, for each DGM structure, we assessed three models, using (1) baseline QSM values only; (2) baseline DGM regional volumes only; and (3) both baseline QSM values and baseline regional volumes as explanatory variables, reporting only the third due to similar effect sizes (model 1 vs. 3, and model 2 vs 3). These longitudinal models were:

Outcome variable at follow‐up = β _o_ + β_1_ (baseline QSM values) + β_2_ (baseline regional volume) + β_3_ (baseline age) + β_4_ (sex) + β_5_ (baseline disease duration) + β_6_ (baseline outcome variable) + β_7_ (follow‐up interval) + ϵ

Results were corrected for multiple comparisons. Each model included QSM values and regional volumes of the four DGM regions across seven outcomes (five clinical outcomes, two MRI parameters for cross‐sectional analyses) for cross‐sectional analyses (marked in Table 1 and Table 2), and five clinical outcomes for longitudinal analyses, yielding 28 models for cross‐sectional and 20 for longitudinal analyses. Statistical significance was determined using 10000 iterations of permutation and a false discovery rate correction (FDR) of 0.05. FDR correction was applied within each group of models sharing the same outcome variables. All analyses were conducted in R v4.4 (R Core Team 2024).

**TABLE 1:** Baseline characteristics of patients.

	Cross‐sectional cohort				
	CIS	RRMS	SPMS	PPMS	Total
N	35	673	26	37	771
Age	39.9 (11.6)	38.9 (10.4)	54.9 (7.9)	51.2 (9.4)	40.0 (11.0)
Sex					
Female (%)	21 (60.0%)	441 (65.2%)	14 (53.8%)	20 (54.1%)	496 (64.3%)
Male (%)	14 (40.0%)	232 (34.5%)	12 (46.2%)	17 (45.9%)	275 (35.7%)
Disease duration (years)	3.0 (2.3)	7.3 (5.7)	21.6 (7.0)	3.4 (3.5)	7.4 (6.3)
QSM values (ppm)					
Thalamus	−0.005 (0.009)	−0.008 (0.010)	−0.011 (0.013)	−0.005 (0.014)	−0.008 (0.010)
Caudate	0.041 (0.019)	0.041 (0.016)	0.057 (0.025)	0.057 (0.026)	0.042 (0.018)
Putamen	0.050 (0.029)	0.048 (0.026)	0.076 (0.030)	0.073 (0.039)	0.050 (0.028)
Pallidum	0.099 (0.032)	0.097 (0.027)	0.120 (0.031)	0.120 (0.038)	0.099 (0.028)
∔	∔		−–	∔	∔
MRI surrogates (mL)					
White matter lesion volume ^a^	0.9 (1.5)	5.8 (8.5)	21.0 (15.6)	9.9 (13.8)	6.3 (9.9)
Normalized total brain volume	1436.7 (49.13)	1421.3 (53.5)	1342.7 (90.2)	1397.0 (51.5)	1418.2 (56.8)
Regional volumes (mL) **^b^**					
Thalamus	13.7 (0.9)	13.0 (1.2)	11.3 (1.6)	12.7(1.3)	13.0 (1.3)
Caudate	6.9 (0.7)	6.6 (0.8)	6.0 (1.0)	6.4 (0.6)	6.6 (0.8)
Putamen	8.9 (0.7)	8.6 (0.9)	7.6 (1.4)	8.3 (0.9)	8.6 (0.9)
Pallidum	3.0 (0.4)	2.9 (0.4)	2.5 (0.6)	2.9 (0.4)	2.9 (0.5)
Disease Modifying Treatment					
Untreated	16 (45.7%)	159 (23.6%)	9 (34.6%)	19 (51.4%)	203 (26.3%)
Cladribine	0 (0.0%)	3 (0.4%)	0 (0.0%)	0 (0.0%)	3 (0.4%)
Dimethyl fumarate	2 (5.7%)	109 (16.2%)	0 (0.0%)	0 (0.0%)	111 (14.4%)
Fingolimod	0 (0.0%)	100 (14.9%)	1 (3.8%)	0 (0.0%)	101 (13.1%)
Glatiramer acetate	9 (25.7%)	90 (13.4%)	0 (0.0%)	0 (0.0%)	99 (12.8%)
Interferon β	8 (22.9%)	82 (12.2%)	7 (26.9%)	0 (0.0%)	97 (12.6%)
Mitoxantrone	0 (0.0%)	0 (0.0%)	2 (7.7%)	0 (0.0%)	2 (0.3%)
Natalizumab	0 (0.0%)	30 (4.5%)	0 (0.0%)	0 (0.0%)	30 (3.9%)
Ocrelizumab	0 (0.0%)	56 (8.3%)	0 (0.0%)	13 (35.1%)	69 (8.9%)
Rituximab	0 (0.0%)	16 (2.4%)	7 (26.9%)	5 (13.5%)	28 (3.6%)
Teriflunomide	0 (0.0%)	28 (4.2%)	0 (0.0%)	0 (0.0%)	28 (3.6%)

*Note*: Values in the table are presented as mean (standard deviation).

^a^ Lesion volumes are reported without log‐transformation.

^b^ Regional volumes are corrected for head size by dividing each regional brain volume by the individual total intracranial volume (TIV), then multiplying by the cohort mean TIV.

Abbreviation: CIS = clinically isolated syndrome, EDSS = Expanded Disability Status Scale, RRMS = relapsing‐remitting multiple sclerosis, SPMS = secondary progressive multiple sclerosis, PPMS = primary progressive multiple sclerosis, QSM = quantitative susceptibility mapping.

**TABLE 2:** Baseline clinical characteristics.

∔	**Cross‐sectional cohort**					**Longitudinal cohort**
∔	CIS	RRMS	SPMS	PPMS	Total	CIS + RRMS
N	25	569	16	30	640	396
EDSS ^a^	1.0 (0, 1.5)	1.5 (0, 2.0)	5.0 (3.5, 6.0)	4.0 (3.0, 4.5)	1.5 (0, 2.0)	1.0 (0, 2.0)
N	17	420	12	9	458	284
T25FW ^a^	3.7 (3.5, 4.3)	4.2 (3.7, 4.7)	6.8 (5.0, 8.8)	5.3 (4.5, 6.7)	4.2 (3.7, 4.7)	4.2 (3.7, 4.6)
N	17	421	7	6	451	284
NHPT ^a^	16.9 (16.2, 18.8)	18.3 (16.8, 19.8)	27.8 (21.8, 29.3)	21.5 (18.7, 30.3)	18.3 (16.9, 19.8)	18.0 (16.5, 19.3)
N	12	337	7	5	361	165
SDMT ^a^	63.0 (61.8, 73.3)	62.0 (54.0, 68.0)	39.0 (35.5, 45.5)	51.0 (47.0, 57.0)	61.0 (54.0, 68.0)	62.0 (57.0, 70.0)
N	17	420	7	4	448	288
FSMC ^a^	26.0 (20.0, 48.0)	37.0 (23.8, 60.0)	70.0 (56.0, 74.5)	61.0 (53.3, 71.8)	37.5 (23.8, 61.0)	34.5 (24.0, 59.3)

*Note*: Values in the table are presented as median (interquartile range).

^a^ Clinical variables were analyzed as outcome variables.

^b^ The follow‐up interval for all clinical variables ranged from 1 to 4.7 years, with a median of 3.7 years.

Abbreviation: CIS = clinically isolated syndrome, EDSS = Expanded Disability Status Scale, FSMC = Fatigue Scale for Motor and Cognitive Functions, NHPT = Nine‐Hole Peg Test, PPMS = primary progressive multiple sclerosis, RRMS = relapsing‐remitting multiple sclerosis, SDMT = Symbol Digit Modalities Test, SPMS = secondary progressive multiple sclerosis, T25FW = Timed 25‐Foot Walk.

Abbreviations: DGM = deep grey matter, EDSS = expanded disability status scale, FLAIR = fluid‐attenuated inversion recovery, FSMC = fatigue scale for motor and cognitive functions, MRI = magnetic resonance imaging, MS = multiple sclerosis, *N* = number (sample size), NHPT = nine‐hole peg test, PMS = progressive MS, SDMT = symbol digit modalities test, T1w = T1‐weighted, T25FW = timed 25‐foot walk.

## 3 Results

### 3.1 Study Participants

**FIGURE 1:**
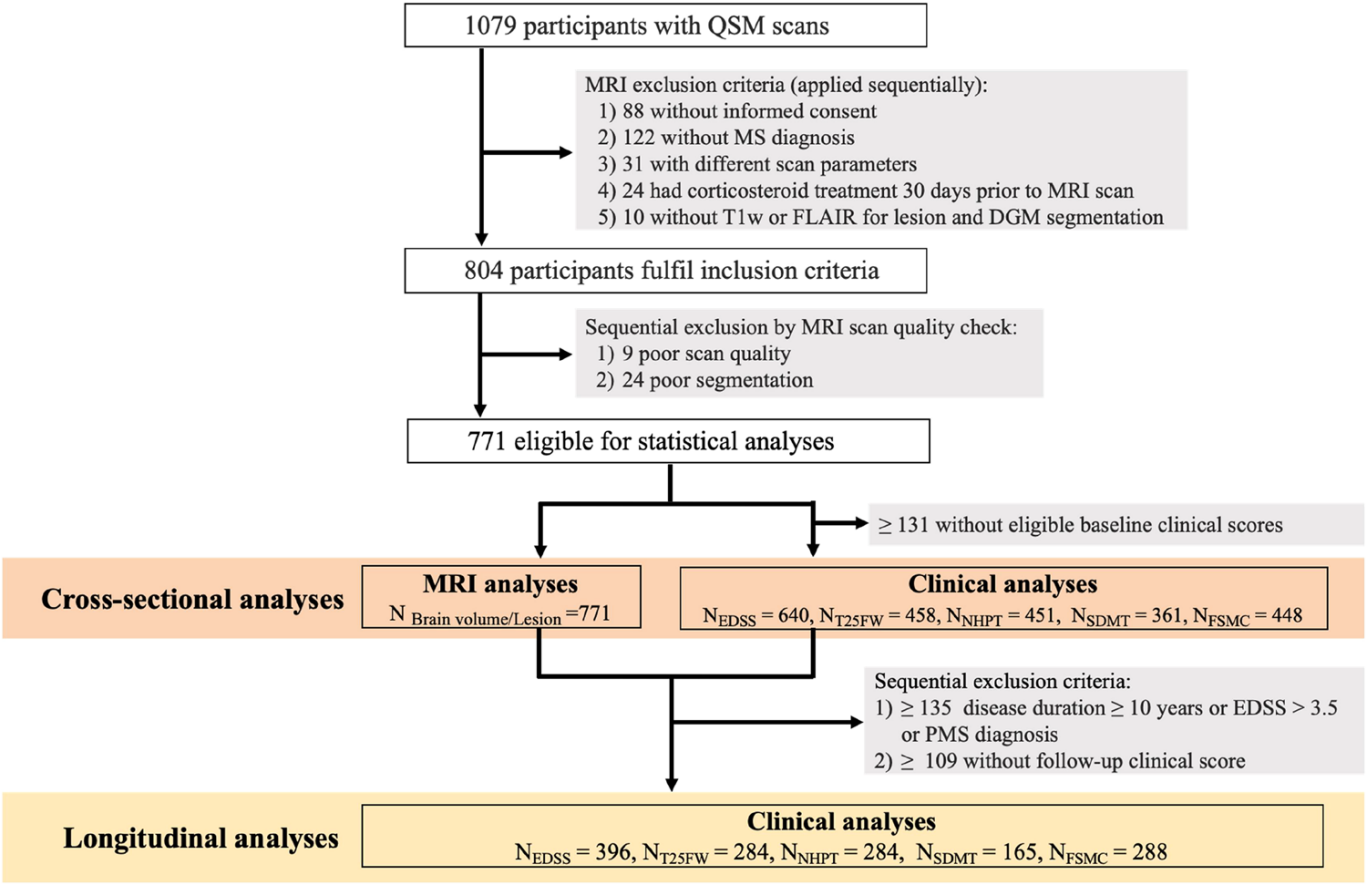
Flow chart for patient selection.

Figure 1 illustrates the patient inclusion and exclusion process. Our study included 771 MS patients, with a mean age of 40 ± 11 years at baseline; 64.3% were female. Baseline MRI scans were obtained between 2019 and 2020, averaging 7.4 years after MS onset. The median follow‐up duration for clinical assessments was 3.7 years. Of the 771 patients, 396 patients had at least one follow‐up clinical score. Patient characteristics are detailed in Tables 1 and 2.

### 3.2 Cross‐Sectional Correlations of all Variables

**FIGURE 2:**
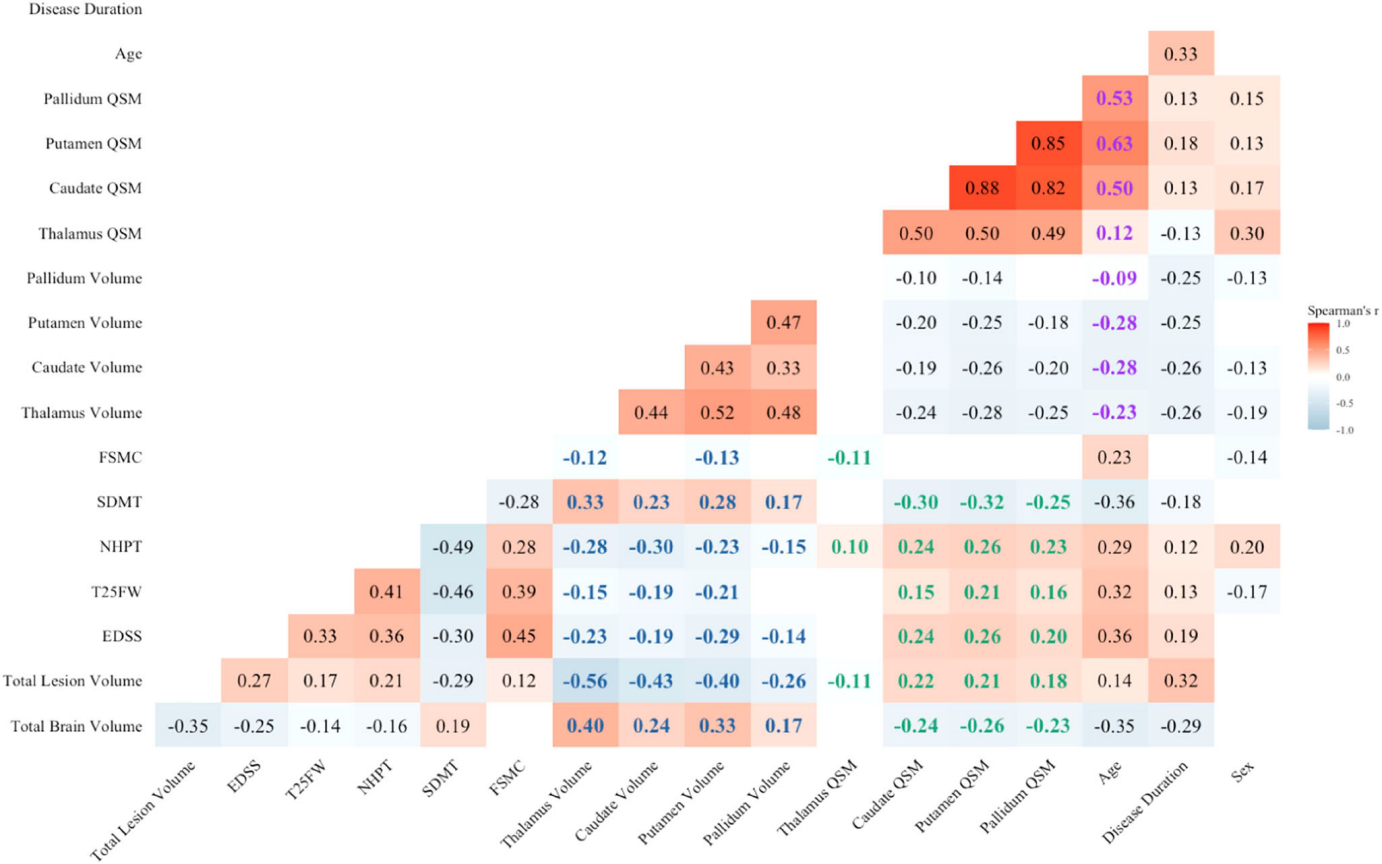
Correlation matrix for all baseline variables. Color cells represent Spearman's correlation coefficients for significant correlations (corrected *p* < 0.05). Correlations of clinical scores and MRI parameters with regional volumes are marked in bold blue and with QSM values in bold green. Correlations of age with regional volumes or with QSM values are marked in bold purple. A complete correlation matrix, including Spearman's correlation coefficients and sample size for all cells, is provided in Supplementary Figure 5. Total lesion volume was logarithmically transformed, sex was coded with female as 0 and male as 1. Abbreviations: EDSS = expanded disability status scale, T25FW = timed 25‐foot walk, NHPT = nine‐hole peg test, SDMT = symbol digit modalities test, FSMC = fatigue scale for motor and cognitive functions, QSM = susceptibility values

Figure 2 shows Spearman's correlations among all variables after FDR corrections. Basal ganglia QSM values demonstrated significant correlations with multiple clinical and MRI variables. Specifically, higher QSM values were associated with lower NBV (Caudate: Spearman correlation coefficients (*r_s_*) = –0.24, *p* = 1.3 × 10^−11^, *p*_corrected_ < 3.9 × 10^−11^; Putamen: *r_s_* = ‐0.26, *p* = 2 × 10^−13^, *p*_corrected_ = 8.9 × 10^−13^; Pallidum: *r_s_* = –0.23, *p* = 1 × 10^−11^, *p*_corrected_ = 2 × 10^−10^) and increased TLV (Caudate: *r_s_* = 0.22, *p* = 1 × 10^−9^, *p*_corrected_ = 3 × 10^−9^; Putamen: *r_s_* = 0.21, *p* = 7 × 10^−9^, *p*_corrected_ = 2 × 10^−8^; Pallidum: *r_s_* = 0.18, *p* = 8 × 10^−7^, *p*_corrected_ = 2 × 10^−6^). Clinically, higher basal ganglia QSM values correlated with increased disability as reflected by higher EDSS scores (Caudate: *r_s_* = 0.24, *p* = 6 × 10^−10^, *p*_corrected_ = 1 × 10^−9^; Putamen: *r_s_* = 0.26, *p* = 2 × 10^−11^, *p*_corrected_ = 7 × 10^−11^; Pallidum: *r_s_* = 0.2, *p* = 2 × 10^−7^, *p*_corrected_ = 4 × 10^−7^), longer completion time for T25FW (Caudate: *r_s_* = 0.15, *p* = 1 × 10^−3^, *p*_corrected_ = 1 × 10^−3^; Putamen: *r_s_* = 0.21, *p* = 8 × 10^−6^, *p*_corrected_ = 1 × 10^−5^; Pallidum: *r_s_* = 0.16, *p* = 5 × 10^−4^, *p*_corrected_ = 8 × 10^−4^), slower performance on NHPT (Caudate: *r_s_* = 0.24, *p* = 3 × 10^−7^, *p*_corrected_ = 5 × 10^−7;^ Putamen: *r_s_* = 0.26, *p* = 3 × 10^−8^, *p*_corrected_ = 7 × 10^−8^; Pallidum: *r_s_* = 0.23, *p* = 1 × 10^−6^, *p*_corrected_ = 2 × 10^−6^), and lower SDMT scores (Caudate: *r_s_* = 0.3, *p* = 8 × 10^−9^, *p*_corrected_ = 2 × 10^−8;^ Putamen: *r_s_* = 0.32, *p* = 3 × 10^−10^, *p*_corrected_ = 7 × 10^−10^; Pallidum: *r_s_* = 0.25, *p* = 2 × 10^−6^, *p*_corrected_ = 3 × 10^−6^), indicating greater motor and cognitive impairment.

Thalamic QSM values exhibited fewer significant correlations. Lower thalamic QSM values were significantly correlated with increased TLV (*r_s_* = –0.11, *p* = 3 × 10^−3^, *p*_corrected_ = 4.1 × 10^−3^), slower NHPT performance (*r_s_* = 0.1, *p* = 3 × 10^−2^, *p*_corrected_ = 3 × 10^−2^), and lower FSMC scores (*r_s_* = –0.11, *p* = 2 × 10^−2^, *p*_corrected_ = 2 × 10^−2^), indicating a relationship between reduced thalamic QSM values and greater lesion burden, impaired upper limb movements, and increased fatigue.

Regional volumes displayed stronger correlations with both clinical and MRI outcomes (absolute *r_s_*: 0.12–0.56) compared to QSM values (absolute *r_s_*: 0.11–0.32). Additionally, basal ganglia QSM values were negatively correlated with DGM volumes. Notably, basal ganglia QSM values showed stronger positive correlations with age (Caudate: *r_s_* = 0.5, *p* < 1 × 10^−16^, *p*_corrected_ < 1 × 10^−16^; Putamen: *r_s_* = 0.63, *p* < 1 × 10^−16^, *p*_corrected_ < 1 × 10^−16^; Pallidum: *r_s_* = 0.53, *p* < 1 × 10^−16^, *p*_corrected_ ≤ 1 × 10^−16^) than with disease duration (Caudate: *r_s_* = 0.13, *p* = 3.3 × 10^−4^, *p*_corrected_ = 4.9 × 10^−4^; Putamen: *r_s_* = 0.18, *p* = 6.3 × 10^−7^, *p*_corrected_ = 1.2 × 10^−6^; Pallidum: *r_s_* = 0.13, *p* = 1.9 × 10^−4^, *p*_corrected_ = 2.9 × 10^−4^).

### 3.3 Cross‐Sectional Associations of DGM QSM Values and Regional Volumes With Clinical and MRI Parameters

**FIGURE 3:**
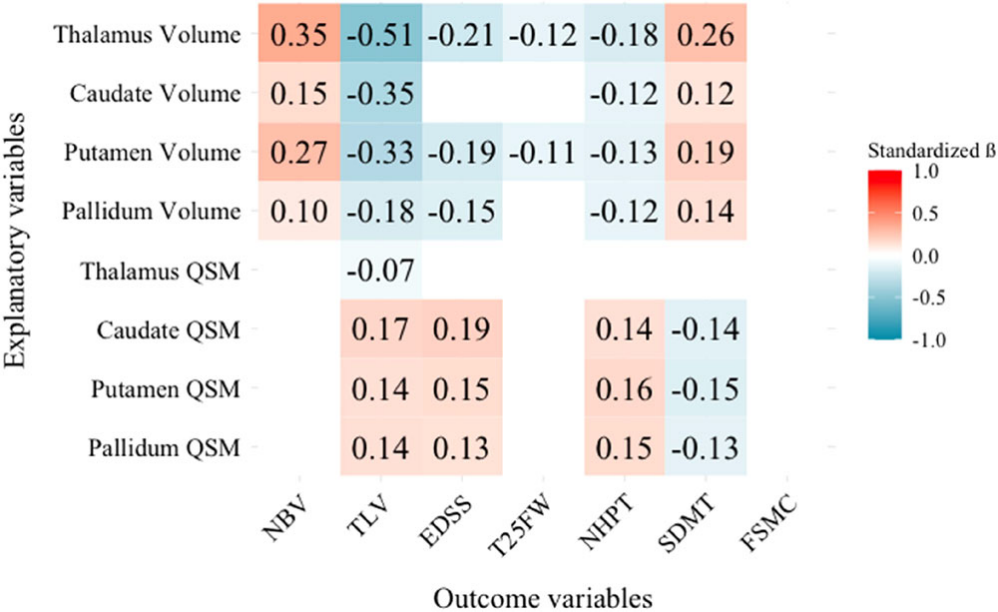
Cross‐sectional associations of DGM susceptibility and volumes with clinical and MRI parameters. Colored cells represent standardized β of significant associations (corrected *p* < 0.05). Each column demonstrates the associations of one outcome variable at baseline with four different regional DGM volumes and QSM values, adjusted for age, sex, and disease duration. Additionally, each regional DGM volume was corrected for its corresponding QSM values and vice versa. A complete version of this figure, including standardized β and sample size for all cells, is provided in Supplementary Figure 7. Abbreviations: EDSS = expanded disability status scale, FSMC = fatigue scale for motor and cognitive functions, NBV = normalized brain volume, NPHT = nine‐hole peg test, QSM = QSM values (susceptibility values), SDMT = symbol digit modalities test, standardized β = Standardized beta coefficient, T25FW = timed 25‐foot walk, TLV = total lesion volume.

Figure 3 illustrates the associations of QSM values and regional volumes (corrected for each other, see 2.4) with clinical and MRI parameters at baseline, adjusting for age, sex, and disease duration. Significant standardized beta values (β), denoting the strength of associations, are given in each cell. In general, regional volumes exhibited stronger significant associations with clinical outcomes and MRI surrogates (absolute β range: 0.10–0.51) than DGM QSM values (absolute β range: 0.07–0.19). Still, high basal ganglia QSM values were significantly associated with lesion burden and poor clinical assessments. Specifically, increased basal ganglia QSM values were significantly associated with increased TLV (Caudate: β = 0.17, *p*_corrected_ < 2 × 10^−16^; Putamen: β = 0.14, *p*_corrected_ < 2 × 10^−16^; Pallidum: β = 0.14, *p*_corrected_ < 2 × 10^−16^), EDSS scores (Caudate: β = 0.19, *p*_corrected_ < 2 × 10^−16^; Putamen: β = 0.15, *p*_corrected_ < 2 × 10^−16^; Pallidum: β = 0.13, *p*_corrected_ < 2 × 10^−16^), and NHPT (Caudate: β = 0.14, *p*_corrected_ = 0.02; Putamen: β = 0.16, *p*_corrected_ = 0.02; Pallidum: β = 0.13, *p*_corrected_ = 0.02). Additionally, basal ganglia QSM values were negatively associated with SDMT (Caudate: β = 0.14, *p*_corrected_ = 0.03; Putamen: β = 0.15, *p*_corrected_ = 0.03; Pallidum: β = 0.13, *p*_corrected_ = 0.04). Thalamic QSM values were only negatively associated with the TLV (β = –0.07, *p* = 0.03), and the strength of association is small. Additional results from regression models that separately link either DGM QSM values or regional volumes to clinical scores and MRI surrogates are reported in Supplementary Figure 6. All cross‐sectional regression models were statistically significant. Their corresponding fit estimates are reported in Supplementary 8.

### 3.4 Longitudinal Associations of DGM QSM Values, Volumes, and Follow‐up Clinical Outcomes

**FIGURE 4:**
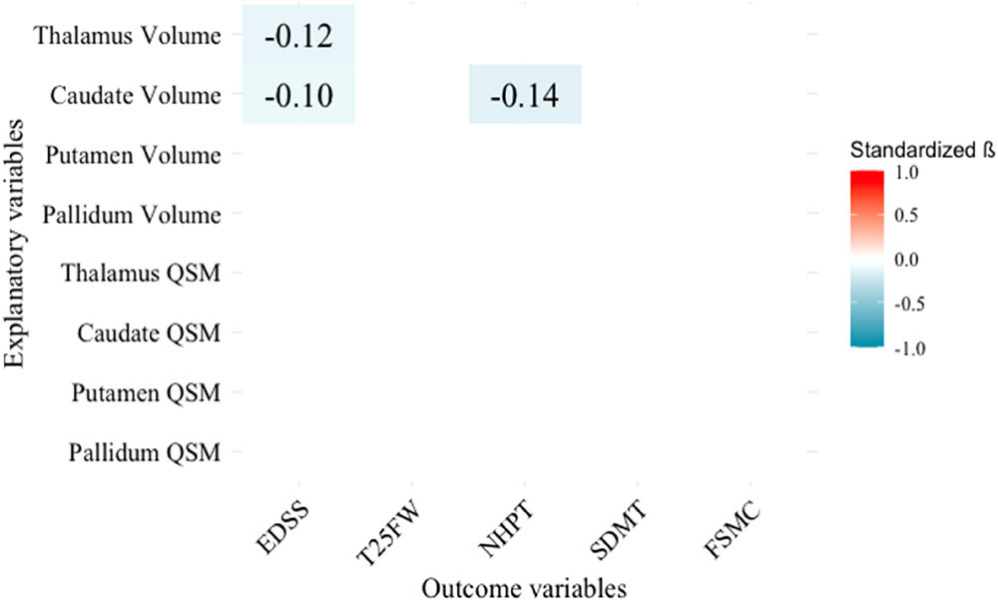
Longitudinal associations of DGM susceptibility and volumes with clinical variables. Colored cells represent standardized β values of significant associations (FDR corrected *p* < 0.05). Each column illustrates the associations of one follow‐up outcome variable with four different regional DGM volumes and QSM values at baseline. The associations were adjusted for age, sex, disease duration, follow‐up interval, and outcome variables at baseline. Additionally, each regional DGM volume was corrected for its corresponding QSM values and vice versa. A complete version of this figure (ß, p, and n in each cell) is provided in Supplementary Figure 9. Abbreviations: EDSS = expanded disability status scale, FSMC = fatigue scale for motor and cognitive functions, NHPT = nine‐hole peg test, QSM = QSM values (susceptibility values), SDMT = symbol digit modalities test, Standardized β = standardized beta coefficient, T25FW = timed 25‐foot walk.

Figure 4 presents the longitudinal associations of baseline DGM QSM values and regional DGM volumes with follow‐up clinical outcomes, adjusting for sex, age, disease duration, intervals, and baseline clinical scores (see Section 2.4 for regression models). The findings revealed no significant association between baseline QSM values and any single follow‐up clinical outcome. However, regional brain volumes, particularly of the thalamus and caudate, were significant predictors of clinical outcomes. Lower baseline thalamic volumes were associated with higher EDSS (β = –0.11, *p*_corrected_ = 0.02). Similarly, lower baseline caudate volumes were associated with decreased EDSS scores (β = –0.10, *p*_corrected_ = 0.04) and with decreased dexterity indicated by higher NPHT scores (β = –0.14, *p*_corrected_ = 0.04). Additional results from regression models that separately link either baseline DGM QSM values or baseline regional volumes to follow‐up clinical scores are reported in Supplementary Figure 10. All longitudinal regression models were statistically significant. Supplementary 11 reports the corresponding fit estimates.

## 4 Discussion

Our study demonstrated cross‐sectional associations between DGM QSM values, clinical outcomes, and MRI surrogates, even after controlling for the confounders of age, sex, and disease duration. However, unlike regional DGM volumes, DGM QSM values did not predict clinical outcomes in our early‐stage MS cohort.

In particular, elevated QSM values in the basal ganglia (caudate, putamen, and pallidum) were significantly associated with several adverse clinical and MRI outcomes. Higher QSM values correlated with increased TLV, greater disabilities measured by EDSS, reduced dexterity reflected by slower NHPT performance, and poorer cognitive function as indicated by decreased SDMT scores. These findings align with our recent meta‐analysis linking higher putamen QSM values to higher EDSS scores (Voon et al. 2024) and extend those associations to the caudate and pallidum. This supports existing evidence that QSM imaging can detect pathological iron accumulation in the basal ganglia, potentially resulting from disrupted iron homeostasis, oxidative injury (Haider et al. 2014), and atrophy‐related iron concentration increase (Schweser et al. 2018, Pontillo et al. 2021).

Our findings showed a significant negative association between basal ganglia QSM values and SDMT scores. This corroborates a previous study by Fujiwara et al. (2017), which reported worsening cognitive function with increased pallidum QSM values. However, we were unable to identify a plausible pattern of associations across DGM structures regarding functional domains of DGM structures and corresponding clinical domains or specific aspects of MS pathology like inflammation (TLV) or brain atrophy (NBV), neither in our data nor in our previous meta‐analysis (Voon et al. 2024). Nevertheless, associations between DGM QSM values and clinical scores, as well as MRI parameters as a whole, are robust, even after adjusting for confounding variables.

In contrast to the basal ganglia, lower thalamic QSM values were linked primarily to adverse MRI outcomes, particularly higher TLV. Additionally, we found that thalamic QSM values positively correlated with age, whereas disease duration showed a negative correlation (Figure 2). Given the complex interplay of thalamic QSM values between MS‐related and age‐related processes on the one hand and the contrary effects of myelin and iron on the other hand, the interpretation of our findings remains speculative. In principle, our observations are compatible with age‐related thalamic iron increase (Hametner et al. 2013) as well as MS‐related thalamic myelin loss (Cagol et al. 2024, Vercellino et al. 2009) and iron depletion (Hametner et al. 2013, Cagol et al. 2024). Histological evidence suggests that iron exerts a stronger effect on QSM values in DGM (Hametner et al. 2013). Prior studies using combined QSM and R2* imaging demonstrated concurrent reductions in both QSM and R2* values in the thalamus of MS patients, indeed suggesting iron loss as the primary driver of reduced QSM values in the thalamus (Schweser et al. 2018, Fujiwara et al. 2017). A plausible pathological mechanism underlying thalamic QSM reduction involves iron depletion following the loss of oligodendrocytes in the thalamic white matter. Hametner et al. (Hametner et al. 2013) demonstrated that chronic inflammation can promote iron release from oligodendrocytes and increase the expression of iron‐exporting proteins, such as ferroxidase hephaestin, contributing to a net loss of iron in normal‐appearing white matter among patients with MS compared to HC (Hametner et al. 2013). A similar process may underlie QSM reductions in the thalamus.

To provide a frame of reference for the effect sizes of QSM values and to account for the confounding influence of DGM volumes, which are related to MS disability (Eshaghi et al. 2018), we analyzed the analogous effect sizes of regional DGM volumes and, to search for independent contributions, set up statistical models including both variables. By replicating established associations of regional DGM volumes (Eshaghi et al. 2018, Hänninen et al. 2020, Magon et al. 2020), we ensure our data's validity and statistical power. Of note, cross‐sectional associations of DGM volumes with both clinical scores and MRI surrogates were generally stronger than those of respective DGM QSM values. This trend continued in the longitudinal analyses, where only DGM volumes demonstrated predictive power for clinical scores.

Our findings also demonstrate that thalamic volume predicts worsening EDSS scores, aligning with previous research (Eshaghi et al. 2018, Magon et al. 2020). Furthermore, thalamic volume exhibited higher standardized beta coefficients than basal ganglia volume in the cross‐sectional regression models, suggesting that thalamic atrophy may be more strongly associated with clinical and MRI measures in MS. This aligns with previous studies highlighting that the thalamus undergoes earlier and more extensive atrophy compared to other DGM structures (Krijnen et al. 2023, Eshaghi et al. 2018, Salman et al. 2025). Several physiological mechanisms may account for this differential vulnerability. The thalamus functions as a central relay hub with extensive connections with the cerebral cortex, cortical, and subcortical regions, making it particularly susceptible to secondary neurodegeneration via Wallerian or upward degeneration (Krijnen et al. 2023, Mahajan et al. 2020). Additionally, the close proximity to the ventricular system exposes the thalamus to cerebrospinal fluid‐borne inflammatory mediators, facilitating periventricular gradient neuroinflammation and subsequent neuronal damage (Magliozzi et al. 2022, Koubiyr et al. 2024). Together, these factors may contribute to the more pronounced and clinically relevant thalamic volume loss observed in MS.

There are still methodological issues with QSM reconstructions, including the lack of a commonly accepted methodological standard (Bilgic et al. 2024), which we addressed by choosing the least artifact‐prone technique out of three different processing pipelines. In addition, physiological aging interferes with DGM QSM values, as illustrated by the finding that simple correlations of basal ganglia QSM values were more strongly correlated with age than with MS disease duration. Furthermore, the age‐dependent trajectories of DGM QSM values seem to be nonlinear in contrast to DGM volumes (Treit et al. 2021), making it more difficult to account for them. Finally, other pathophysiological factors apart from iron accumulation and myelin loss may play a role in the DGM QSM change in MS. For example, an early animal study demonstrated iron depletion in a relatively short time under specific pathological conditions (Hill 1985).

We acknowledge the limitations of our study. First, the relatively short follow‐up period for clinical scores may have limited the statistical power regarding the associations between DGM QSM values and clinical progression over time. Second, the longitudinal analyses were restricted to patients in early‐stage MS, limiting our findings' generalizability to longer disease durations or more advanced disability. Third, our analysis of the thalamus as a single structure does not account for the heterogeneity of thalamic subnuclei, each with distinct cytoarchitecture and myelin content, which may contribute differently to QSM values and their associations with clinical scores. Fourth, the absence of a healthy control group prevents us from distinguishing QSM changes due to normal aging from those driven by MS pathology. Fifth, we did not account for the potential influence of disease‐modifying treatment, which might influence both iron metabolism and neurodegeneration. Finally, although some longitudinal QSM‐clinical associations reached statistical significance, their small effect sizes raise concerns about their clinical relevance.

Overall, it seems that DGM QSM values are a promising research measure for studying the pathophysiology of MS, but the precise biological underpinnings, including their temporal kinetics, need to be elucidated. At present, the prognostic value of DGM QSM at the individual level, particularly in early‐stage MS, seems out of sight. Future studies should investigate the predictive utility of QSM values across a broader range of disease stages (i.e., CIS, RRMS, PMS), ideally over longer follow‐up periods.

## 5 Conclusion

DGM QSM values are robustly associated with MS severity, and the MRI surrogate of TLV cross‐sectionally. The association with iron accumulation and myelin loss makes these measures an attractive tool for studying the pathophysiology of MS. However, the value of their use in clinical routines in early MS is not yet in sight.

## Author Contributions

**Cui Ci Voon**: conceptualization, investigation, writing—original draft, visualization, methodology, writing—review and editing. **Jakob Meineke**: methodology, validation, writing—review and editing. **Tun Wiltgen**: methodology, data curation, writing—review and editing. **Julian Mcginnis**: methodology, data curation, writing—review and editing. **Ronja Berg**: methodology, data curation, writing—review and editing. **Christine Preibisch**: methodology, data curation, writing—review and editing. **Sarah Schlaeger**: writing—review and editing. **Benedikt Wiestler**: supervision, data curation, writing—review and editing. **Christina Engl**: data curation, writing—review and editing. **Achim Berthele**: supervision, writing—review and editing. **Jan S. Kirschke**: writing—review and editing, supervision, data curation. **Bernhard Hemmer**: supervision, writing—review and editing. **Mark Mühlau**: funding acquisition, conceptualization, writing—original draft, writing—review and editing, supervision, methodology.

## Ethical Contributions

The Ethics Committee at Technical University Munich approved our project (approval no.: 2019–83_1‐S‐SR, 25^th^ May 2022).

## Consent

Patients provided written informed consent for the scientific use of data obtained in clinical routine.

## Conflicts of Interest

The authors declared the following potential conflict of interest with respect to research, authorship, and/or publication of this article: CE received financial support for attending ECTRIMS 2024 from Sanofi (conference registration fees, travel and accomodation expenses). She received compensation for preparing a lecture for a planned symposium, which was later canceled from Roche. AB receives funding from the Innovationsausschuss of the German Federal Joint Committee (G‐BA; grant 01VSF23040) and from the German Federal Ministry of Education and Research (BMBF; grant 01ZZ2102B). He has received consulting and/or speaker fees from Alexion, Argenx, Biogen, Horizon/Amgen, Merck, Novartis, Roche and Sandoz/Hexal, and his institution has received compensation for clinical trials from Alexion, Biogen, Merck, Novartis, Roche, and Sanofi Genzyme; all outside the present work. BH is associated with DIFUTURE (Data Integration for Future Medicine) [BMBF 01ZZ1804[A‐I]]. He received funding for the study by the European Union's Horizon 2020 Research and Innovation Program [grant MultipleMS, EU RIA 733161,; WISDOM RIA 101137154], the Bundesministerium für Bildung und Forschung [Clinspect‐M consortium] and the Deutsche Forschungsgemeinschaft (DFG, German Research Foundation) under Germany's Excellence Strategy within the framework of the Munich Cluster for Systems Neurology [EXC 2145 SyNergy—ID 390857198]. He has served on scientific advisory boards for Novartis, Hoffman LaRoche and Polpharma; he has served as DMSC member for AllergyCare, Sandoz, Polpharma, Biocon and TG therapeutics; his institution received research grants from Roche for multiple sclerosis research. He has received honoraria for counseling (Gerson Lehrmann Group). The other authors report no conflicts of interest related to this study.

## Peer Review

The peer review history for this article is available at https://publons.com/publon/10.1002/brb3.70988

## Supporting information




**Supplementary Materials**: brb370988‐sup‐0001‐SuppMat.docx

## Data Availability

The data that support the findings of this study are available from the corresponding author upon reasonable request.

## References

[brb370988-bib-0002] Boehm, C. , N. Sollmann , J. Meineke , et al. 2022. “Preconditioned Water‐Fat Total Field Inversion: Application to Spine Quantitative Susceptibility Mapping.” Magnetic Resonance in Medicine 87, no. 1: 417–430. 10.1002/mrm.28903. 34255370

[brb370988-bib-0003] Burgetova, A. , P. Dusek , M. Vaneckova , et al. 2017. “Thalamic Iron Differentiates Primary‐Progressive and Relapsing‐Remitting Multiple Sclerosis.” Ajnr American Journal of Neuroradiology 38, no. 6: 1079–1086. 10.3174/ajnr.A5166. 28450431 PMC7960078

[brb370988-bib-0004] Cagol, A. , M. Ocampo‐Pineda , P.‐J. Lu , et al. 2024. “Advanced Quantitative MRI Unveils Microstructural Thalamic Changes Reflecting Disease Progression in Multiple Sclerosis.” Neurol Neuroimmunol Neuroinflamm 11, no. 6: e200299. 10.1212/NXI.0000000000200299. 39270143 PMC11409727

[brb370988-bib-0005] Chiang, G. C. , J. Hu , E. Morris , et al. 2018. “Quantitative Susceptibility Mapping of the Thalamus: Relationships With Thalamic Volume, Total Gray Matter Volume, and T2 Lesion Burden.” Ajnr American Journal of Neuroradiology 39, no. 3: 467–472. 10.3174/ajnr.A5537. 29371258 PMC6060040

[brb370988-bib-0006] De Lury, A. D. , J. A. Bisulca , J. S. Lee , et al. 2023. “Magnetic Resonance Imaging Detection of Deep Gray Matter Iron Deposition in Multiple Sclerosis: A Systematic Review.” Journal of the Neurological Sciences 453: 120816. 10.1016/j.jns.2023.120816. 37827008

[brb370988-bib-0007] De Meo, E. , L. Storelli , L. Moiola , et al. 2021. “ *In Vivo* Gradients of Thalamic Damage in Paediatric Multiple Sclerosis: A Window into Pathology.” Brain 144, no. 1: 186–197. 10.1093/brain/awaa379. 33221873

[brb370988-bib-0008] Eshaghi, A. , F. Prados , W. J. Brownlee , et al. 2018. “Deep Gray Matter Volume Loss Drives Disability Worsening in Multiple Sclerosis.” Annals of Neurology 83, no. 2: 210–222. 10.1002/ana.25145. 29331092 PMC5838522

[brb370988-bib-0009] Fujiwara, E. , J. A. Kmech , D. Cobzas , et al. 2017. “Cognitive Implications of Deep Gray Matter Iron in Multiple Sclerosis.” Ajnr American Journal of Neuroradiology 38, no. 5: 942–948. 10.3174/ajnr.A5109. 28232497 PMC7960387

[brb370988-bib-0010] Hagemeier, J. , R. Zivadinov , M. G. Dwyer , et al. 2018. “Changes of Deep Gray Matter Magnetic Susceptibility Over 2 Years in Multiple Sclerosis and Healthy Control Brain.” NeuroImage: Clinical 18: 1007–1016. 10.1016/j.nicl.2017.04.008. 29868452 PMC5984575

[brb370988-bib-0011] Haider, L. , C. Simeonidou , G. Steinberger , et al. 2014. “Multiple Sclerosis Deep Grey Matter: The Relation Between Demyelination, Neurodegeneration, Inflammation and Iron.” Journal of Neurology, Neurosurgery, and Psychiatry 85, no. 12: 1386–1395. 10.1136/jnnp-2014-307712. 24899728 PMC4251183

[brb370988-bib-0012] Hallgren, B. , and P. Sourander . 1958. “The Effect of Age on the Non‐Haemin Iron in the Human Brain.” Journal of Neurochemistry 3, no. 1: 41–51. 10.1111/j.1471-4159.1958.tb12607.x. 13611557

[brb370988-bib-0013] Hametner, S. , I. Wimmer , L. Haider , et al. 2013. “Iron and Neurodegeneration in the Multiple Sclerosis Brain.” Annals of Neurology 74, no. 6: 848–861. 10.1002/ana.23974. 23868451 PMC4223935

[brb370988-bib-0014] Hänninen, K. , M. Viitala , T. Paavilainen , et al. 2020. “Thalamic Atrophy Predicts 5‐Year Disability Progression in Multiple Sclerosis.” Frontiers in Neurology 11: 606. 10.3389/fneur.2020.00606. 32760339 PMC7373757

[brb370988-bib-0015] Hidalgo De, L.a M. Cruz , P. Valsasina , S. Mesaros , et al. 2021. “Clinical Predictivity of Thalamic Sub‐Regional Connectivity in Clinically Isolated Syndrome: A 7‐Year Study.” Molecular Psychiatry 26: 2163–2174. 10.1038/s41380-020-0726-4. 32322087

[brb370988-bib-0016] Hill, J. M. 1985. “Iron Concentration Reduced in Ventral Pallidum, Globus Pallidus, and Substantia Nigra by GABA‐Transaminase Inhibitor, Gamma‐Vinyl GABA.” Brain Research 342, no. 1: 18–25. 10.1016/0006-8993(85)91348-4. 4041811

[brb370988-bib-0017] Keihaninejad, S. , R. A. Heckemann , G. Fagiolo , et al. 2010. “A Robust Method to Estimate the Intracranial Volume Across MRI Field Strengths (1.5T and 3T).” Neuroimage 50, no. 4: 1427–1437. 10.1016/j.neuroimage.2010.01.064. 20114082 PMC2883144

[brb370988-bib-0018] Kipp, M. , N. Wagenknecht , C. Beyer , et al. 2015. “Thalamus Pathology in Multiple Sclerosis: From Biology to Clinical Application.” Cellular and Molecular Life Sciences 72: 1127–1147. 10.1007/s00018-014-1787-9. 25417212 PMC11113280

[brb370988-bib-0019] Koubiyr, I. , T. Yamamoto , S. Blyau , et al. 2024. “Vulnerability of Thalamic Nuclei at CSF Interface During the Entire Course of Multiple Sclerosis.” Neurology Neuroimmunology and Neuroinflammation 11, no. 3: e200222. 10.1212/NXI.0000000000200222. 38635941 PMC11087027

[brb370988-bib-0020] Krijnen, E. A. , A. W. Russo , and E. Salim Karam , et al. 2023. “Detection of Grey Matter Microstructural Substrates of Neurodegeneration in Multiple Sclerosis.” Brain Communications 5, no. 3: fcad153. 10.1093/braincomms/fcad153. 37274832 PMC10233898

[brb370988-bib-0021] Kurtzke, J. F. 1983. “Rating Neurologic Impairment in Multiple Sclerosis: An Expanded Disability Status Scale (EDSS).” Neurology 33, no. 11: 1444. 10.1212/WNL.33.11.1444. 6685237

[brb370988-bib-0022] Langkammer, C. , T. Liu , M. Khalil , et al. 2013. “Quantitative Susceptibility Mapping in Multiple Sclerosis.” Radiology 267, no. 2: 551–559. 10.1148/radiol.12120707. 23315661 PMC3632806

[brb370988-bib-0023] Leray, E. , J. Yaouanq , and E. Le Page , et al. 2010. “Evidence for a Two‐Stage Disability Progression in Multiple Sclerosis.” Brain 133, no. 7: 1900–1913. 10.1093/brain/awq076. 20423930 PMC2892936

[brb370988-bib-0024] Li, W. , N. Wang , F. Yu , et al. 2015. “A Method for Estimating and Removing Streaking Artifacts in Quantitative Susceptibility Mapping.” Neuroimage 108: 111–122. 10.1016/j.neuroimage.2014.12.043. 25536496 PMC4406048

[brb370988-bib-0025] Liu, T. , W. Y. Xu , P. Spincemaille , et al. 2012. “Accuracy of the Morphology Enabled Dipole Inversion (MEDI) Algorithm for Quantitative Susceptibility Mapping in MRI.” IEEE Transactions on Medical Imaging 31, no. 3: 816–824. 10.1109/TMI.2011.2182523. 22231170 PMC3613569

[brb370988-bib-0026] Liu, Z. , Y. Kee , D. Zhou , et al. 2017. “Preconditioned Total Field Inversion (TFI) Method For Quantitative Susceptibility Mapping.” Magnetic Resonance in Medicine 78, no. 1: 303–315. 10.1002/mrm.26331. 27464893 PMC5274595

[brb370988-bib-0027] Madden, D. J. , and J. L. Merenstein . 2023. “Quantitative Susceptibility Mapping of Brain Iron in Healthy Aging and Cognition.” Neuroimage 282: 120401. 10.1016/j.neuroimage.2023.120401. 37802405 PMC10797559

[brb370988-bib-0028] Magliozzi, R. , G. Fadda , R. A. Brown , et al. 2022. ““Ependymal‐in” Gradient of Thalamic Damage in Progressive Multiple Sclerosis.” Annals of Neurology 92, no. 4: 670–685. 10.1002/ana.26448. 35748636 PMC9796378

[brb370988-bib-0029] Magon, S. , C. Tsagkas , L. Gaetano , et al. 2020. “Volume Loss in the Deep Gray Matter and Thalamic Subnuclei: A Longitudinal Study on Disability Progression in Multiple Sclerosis.” Journal of Neurology 267: 1536–1546. 10.1007/s00415-020-09740-4. 32040710

[brb370988-bib-0030] Mahajan, K. R. , K. Nakamura , J. A. Cohen , et al. 2020. “Intrinsic and Extrinsic Mechanisms of Thalamic Pathology in Multiple Sclerosis.” Annals of Neurology 88, no. 1: 81–92. 10.1002/ana.25743. 32286701 PMC8291218

[brb370988-bib-0031] Mathiowetz, V. , K. Weber , N. Kashman , and G. Volland . 1985. “Adult Norms for the Nine Hole Peg Test of Finger Dexterity.” The Occupational Therapy Journal of Research 5, no. 1: 24–38. 10.1177/153944928500500102.

[brb370988-bib-0032] Mesaros, S. , M. A. Rocca , M. Absinta , et al. 2008. “Evidence of Thalamic Gray Matter Loss in Pediatric Multiple Sclerosis.” Neurology 70: 1107–1112. 10.1212/01.wnl.0000291010.54692.85. 18272867

[brb370988-bib-0033] Motl, R. W. , J. A. Cohen , R. Benedict , et al. 2017. “Validity of the Timed 25‐Foot Walk as an Ambulatory Performance Outcome Measure for Multiple Sclerosis.” Multiple Sclerosis 23, no. 5: 704–710. 10.1177/1352458517690823. 28206828 PMC5405807

[brb370988-bib-0034] Noseworthy, J. H. , and P. Kirkpatrick . 2005. “Natalizumab.” Nature Reviews Drug Discovery 4: 101–102. 10.1038/nrd1637. 15756757

[brb370988-bib-0035] O'brien, R. M. 2007. “A Caution Regarding Rules of Thumb for Variance Inflation Factors.” Quality and Quantity 41: 673–690. 10.1007/s11135-006-9018-6.

[brb370988-bib-0036] Patenaude, B. , S. M. Smith , Kennedy , N. David , and M. Jenkinson . 2011. “A Bayesian Model of Shape and Appearance for Subcortical Brain Segmentation.” Neuroimage 56, no. 3: 907–922. 10.1016/j.neuroimage.2011.02.046. 21352927 PMC3417233

[brb370988-bib-0037] Penner, I. , C. Raselli , M. Stöcklin , et al. 2009. “The Fatigue Scale for Motor and Cognitive Functions (FSMC): Validation of a New Instrument to Assess Multiple Sclerosis‐Related Fatigue.” Multiple Sclerosis 15, no. 12: 1509–1517. 10.1177/1352458509348519. 19995840

[brb370988-bib-0038] Pontillo, G. , S. Cocozza , R. Lanzillo , et al. 2019. “Determinants of Deep Gray Matter Atrophy in Multiple Sclerosis: A Multimodal MRI Study.” Ajnr American Journal of Neuroradiology 40, no. 1: 99–106. 10.3174/ajnr.A5915. 30573464 PMC7048598

[brb370988-bib-0039] Pontillo, G. , M. Petracca , S. Monti , et al. 2021. “Unraveling Deep Gray Matter Atrophy and Iron and Myelin Changes in Multiple Sclerosis.” Ajnr American Journal of Neuroradiology 42, no. 7: 1223–1230. 10.3174/ajnr.A7093. 33888456 PMC8324266

[brb370988-bib-0001] QSM Consensus Organization Committee . Bilgic, B. , and M. Costagli , et al. 2024. “Recommended Implementation of Quantitative Susceptibility Mapping for Clinical Research in the Brain: A consensus of the ismrm Electro‐Magnetic Tissue Properties Study Group.” Magnetic Resonance in Medicine 91, no. 5: 1834–1862. 10.1002/mrm.30006. 38247051 PMC10950544

[brb370988-bib-0040] R Core Team . 2024. R: A Language and Environment for Statistical Computing.

[brb370988-bib-0041] Salman, F. , N. Bergsland , M. G. Dwyer , et al. 2025. “Thalamic Iron in Multiple Sclerosis: Waning Support For the Early‐Rise Late‐Decline Hypothesis.” NeuroImage: Clinical 46: 103771. 10.1016/j.nicl.2025.103771. 40187193 PMC12002950

[brb370988-bib-0042] Schweser, F. , A. L. Raffaini Duarte Martins , and J. Hagemeier , et al. 2018. “Mapping of Thalamic Magnetic Susceptibility in Multiple Sclerosis Indicates Decreasing Iron With Disease Duration: A Proposed Mechanistic Relationship Between Inflammation And Oligodendrocyte Vitality.” Neuroimage 167: 438–452. 10.1016/j.neuroimage.2017.10.063. 29097315 PMC5845810

[brb370988-bib-0043] Smith, A. 1982. Symbol Digit Modalities Test: Manual. Western Psychological Services.

[brb370988-bib-0044] Smith, S. M. , Y. Zhang , M. Jenkinson , et al. 2002. “Accurate, Robust, and Automated Longitudinal and Cross‐Sectional Brain Change Analysis.” Neuroimage 17, no. 1: 479–489. 10.1006/nimg.2002.1040. 12482100

[brb370988-bib-0045] Sun, H. , A. J. Walsh , R. M. Lebel , et al. 2015. “Validation Of Quantitative Susceptibility Mapping With Perls' Iron Staining For Subcortical Gray Matter.” Neuroimage 105: 486–492. 10.1016/j.neuroimage.2014.11.010. 25462797

[brb370988-bib-0046] Thompson, A. J. , B. L. Banwell , F. Barkhof , et al. 2018. “Diagnosis Of Multiple Sclerosis: 2017 Revisions of the Mcdonald Criteria.” The Lancet Neurology 17, no. 2: 162–173. 10.1016/S1474-4422(17)30470-2. 29275977

[brb370988-bib-0047] Treit, S. , N. Naji , P. Seres , et al. 2021. “ r2 * and Quantitative Susceptibility Mapping in Deep Gray Matter Of 498 Healthy Controls From 5 To 90 Years.” Human Brain Mapping 42, no. 14: 4597–4610. 10.1002/hbm.25569. 34184808 PMC8410539

[brb370988-bib-0048] Vercellino, M. , S. Masera , M. Lorenzatti , et al. 2009. “Demyelination, Inflammation, and Neurodegeneration in Multiple Sclerosis Deep Gray Matter.” Journal of Neuropathology and Experimental Neurology 68, no. 5: 489–502. 10.1097/NEN.0b013e3181a19a5a. 19525897

[brb370988-bib-0049] Voon, C. C. , T. Wiltgen , B. Wiestler , et al. 2024. “Quantitative Susceptibility Mapping in Multiple Sclerosis: A Systematic Review and Meta‐Analysis.” NeuroImage: Clinical 42: 103598. 10.1016/j.nicl.2024.103598. 38582068 PMC11002889

[brb370988-bib-0050] Wen, Y. , P. Spincemaille , T. Nguyen , et al. 2021. “Multiecho Complex Total Field Inversion Method (Mctfi) For Improved Signal Modeling in Quantitative Susceptibility Mapping.” Magnetic Resonance in Medicine 86, no. 4: 2165–2178. 10.1002/mrm.28814. 34028868

[brb370988-bib-0051] Wiltgen, T. , J. McGinnis , S. Schlaeger , et al. 2024. “LST‐AI: A Deep Learning Ensemble For Accurate MS Lesion Segmentation.” NeuroImage: Clinical 42: 103611. 10.1016/j.nicl.2024.103611. 38703470 PMC11088188

[brb370988-bib-0052] Wisnieff, C. , S. Ramanan , J. Olesik , et al. 2015. “Quantitative Susceptibility Mapping (QSM) of White Matter Multiple Sclerosis Lesions: Interpreting Positive Susceptibility and the Presence Of Iron.” Magnetic Resonance in Medicine 74, no. 2: 564–570. 10.1002/mrm.25420. 25137340 PMC4333139

[brb370988-bib-0053] Yan, Z. , H. Liu , X. Chen , et al. 2021. “Quantitative Susceptibility Mapping‐Derived Radiomic Features in Discriminating Multiple Sclerosis From Neuromyelitis Optica Spectrum Disorder.” Frontiers in Neuroscience 15: 765634. 10.3389/fnins.2021.765634. 34924934 PMC8678528

[brb370988-bib-0054] Zivadinov, R. , E. Tavazzi , N. Bergsland , et al. 2018. “Brain Iron at Quantitative MRI Is Associated With Disability in Multiple Sclerosis.” Radiology 289, no. 2: 487–496. 10.1148/radiol.2018180136. 30015589 PMC6219694

