# Supplementary material for: Quantitative Susceptibility Mapping of Deep Grey Matter in MS: Association With Clinical Scores and Brain Volume Measures
Source: Brain Behav. 2025 Oct 20;15(10):e70988. doi: 10.1002/brb3.70988 (PMC12537832; doi:10.1002/brb3.70988)
Supplement: Supplementary file 1 — Supplementary Materials: brb370988‐sup‐0001‐SuppMat.docx [file BRB3-15-e70988-s001.docx]

Supplementary Information

**Quantitative susceptibility mapping of deep grey matter in MS: association with clinical scores and brain volume measures**

# Section 1 Multiecho complex total-field inversion (mTFI) processing details

Quantitative susceptibility mapping (QSM) images were reconstructed directly from the multiecho complex images (see Boehm et al. [1] and Wen et al. [2] for related approaches). The inverse problem was modeled according to the equation:

$$\chi={argmin}_{\chi} \sum_{j} \left\| M_{j}(e^{i\gamma B_{0}T_{E,j}D\star\chi}e^{i\Phi_{0}}-e^{i\Phi_{j}}) \right\|_{2}^{2}+\lambda TV_{\nabla M}\left( \chi\right)$$

where $\chi$ is the magnetic susceptibility, $M_{j}$ is the measured image magnitude for echo $j$, $\gamma$ is the gyromagnetic ratio, $B_{0}$ is the external magnetic field generated by the scanner, $TE_{j}$ is the echo time for echo $j$, $D$ is the unit magnetic dipole operator, $\Phi_{0}$ is the phase-offset, and $\Phi_{j}$ is the measured image phase at echo $j$. The final term $TV_{\nabla M}\left( \chi\right)$ represents regularization by Total-Variation, with weights derived from the gradient $\nabla M$ of the echo time weighted image magnitudes, similar to MEDI [3] and scaled by the regularization parameter $\lambda$. The nonlinear formulation ensures correct modeling of the complex measurement noise [2]. Furthermore, systematic errors from intermediate steps such as field mapping and background-field removal [4] are reduced by directly modeling the mapping from susceptibility to the complex multiecho data. The non-convex optimization problem was initialized using the solution from linear Total-Field Inversion [5] and solved iteratively using linearization around the current solution and the conjugate-gradient method.

# Section 2 Evaluation and comparison of processing pipelines

As recommended by the QSM Consensus Organization Committee (2024), the reconstruction of QSM involves crucial processes to ensure the robustness of QSM images:

1) Combination of phase data from multi-channel coils.

2) Unwrapping of channel-combined phase data.

3) Removal of background field inhomogeneities.

4) Generation of brain masks.

5) Estimation of voxel-wise magnetic susceptibility (QSM values) through dipole inversion.

6) Accurate referencing of QSM values.

Many approaches and algorithms have been proposed for each process. To ensure the robustness of our QSM pipeline, we compared our mTFI pipeline with two other established pipelines, which are STI-iLSQR (v3.0_05/2017; https://people.eecs.berkeley.edu/~chunlei.liu/software.html), and MEDI toolbox (version 11/2017; http://pre.weill.cornell.edu/mri/pages/qsm.html). The details of these pipeline comparisons are summarized in Supplementary Table 1.

All multiecho complex images were acquired from the same scanner; they were reconstructed using Compressed SENSE [6] on the scanner and exported to NIFTI format. To compare QSM images and values across different reconstruction pipelines, the same brain mask and reference region were used. Cerebral spinal fluid (CSF) was used as reference for QSM values as reported by Wang et al. [7] while brain masks were calculated based on the last magnitude image, and residual holes in the brain mask were filled as reported in Berg et al. [8]. QSM images derived from the three different pipelines were visually examined. Supplementary Figure 1, 2, and 3, display the coronal, sagittal, and axial planes of QSM images from five randomly selected patients (A-E).

Based on visual inspection, the mcTFI algorithm produced better QSM images by effectively suppressing streaking artifacts and vein-related artifacts compared to the MEDI algorithm. While the iLSQR pipeline also reduced streaking artifacts, it exhibited more shadowing artifacts, as indicated by yellow arrows in Supplementary Figures 1-3. Finally, even though the same mask was used, the mTFI algorithm allowed better preservation of brain surface boundaries in the QSM images compared to the other two QSM reconstruction pipelines. The difference in brain surface delination is indicated by the orange arrows in Supplementary Figures 2 and 3. Due to its superior ability to suppress streaking and shadowing artifacts, the mcTFI algorithm was selected as the QSM reconstruction pipeline for this study. Examples of the excluded QSM images generated from mTFI pipeline, along with the reasons for exclusion were provided in Supplementary Figure 4.

Supplementary Table 1 Summary of QSM pipeline evaluations

| Processing Steps | MEDI | STI - iLSQR | mTFI |
| --- | --- | --- | --- |
| Multi-channel coil-combination | Compressed SENSE | Compressed SENSE | Compressed SENSE |
| Echo-combination/ Field-Mapping | Nonlinearly fitting the complex signal over TEs | Weighting and averaging over TEs | --- |
| Phase unwrapping | Laplacian-based | Laplacian-based | Region-Growing Algorithm ^a^ |
| Brain mask creation | last echo magnitude image | last echo magnitude image | last echo magnitude image |
| Background field removal | Projection onto Dipole Fields | V-SHARP | implicit in TFI |
| Dipole inversion | Morphology enabled dipole inversion (MEDI) | STI iterative least squares algorithm (STI- iLSQR) | multiecho complex total field inversion (mTFI) |
| Referencing | CSF | CSF | CSF |
| Parameters | Spherical mean value (SMV)^b^ = 3  Regularization parameter = 1000 | SMV = 12 | $\lambda=5e^{-5}$ |

^a^ Region- growing algorithm was only used for initialization

^b^ Additional SMW filtering applied to MEDI pipeline

## Supplementary Figure 1

| **MEDI** | **iLQSR** | **mTFI** | **T1w + DGM Mask** |
| --- | --- | --- | --- |


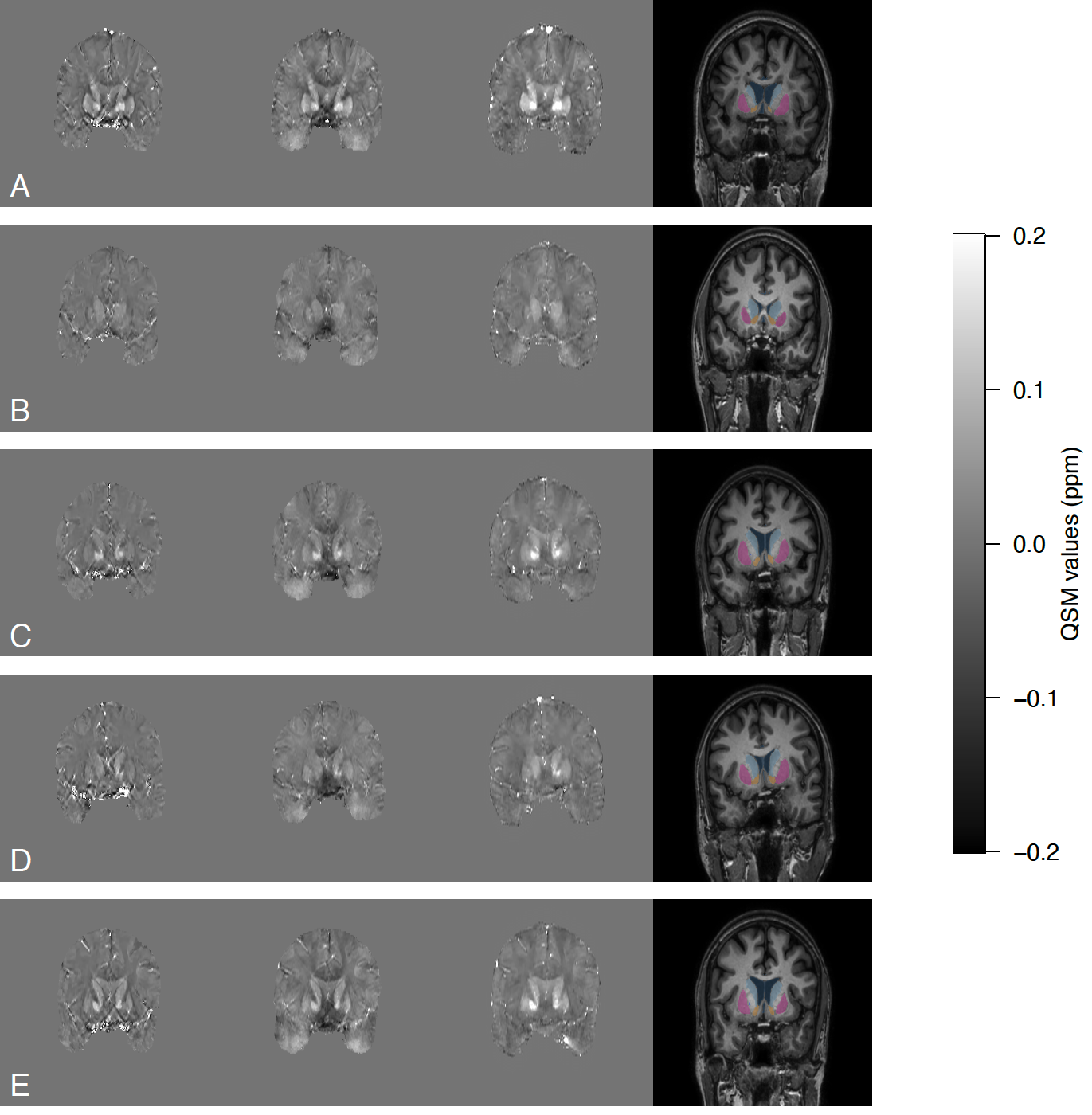


Coronal plane comparison of QSM image

QSM images from three reconstruction pipelines (left to right: MEDI, STI-iLSQR, and mTFI) are compared. T1-weighted images are shown on the right for reference. Each row (A-E) displays images from one randomly selected patient.

**↑** Red arrows indicate streaking artifacts and bright artifacts from veins.

**↑** Yellow arrows indicate shadowing artifacts.

Abbreviations: DGM = deep grey matter; MEDI = Morphology Enabled Dipole Inversion; STI-iLSQR = STI-iterative least squares method; mTFI = multiecho Complex Total Field Inversion, T1w = T1-weighted

## Supplementary Figure 2

| **MEDI** | **iLSQR** | **mTFI** | **T1w + DGM Mask** |
| --- | --- | --- | --- |


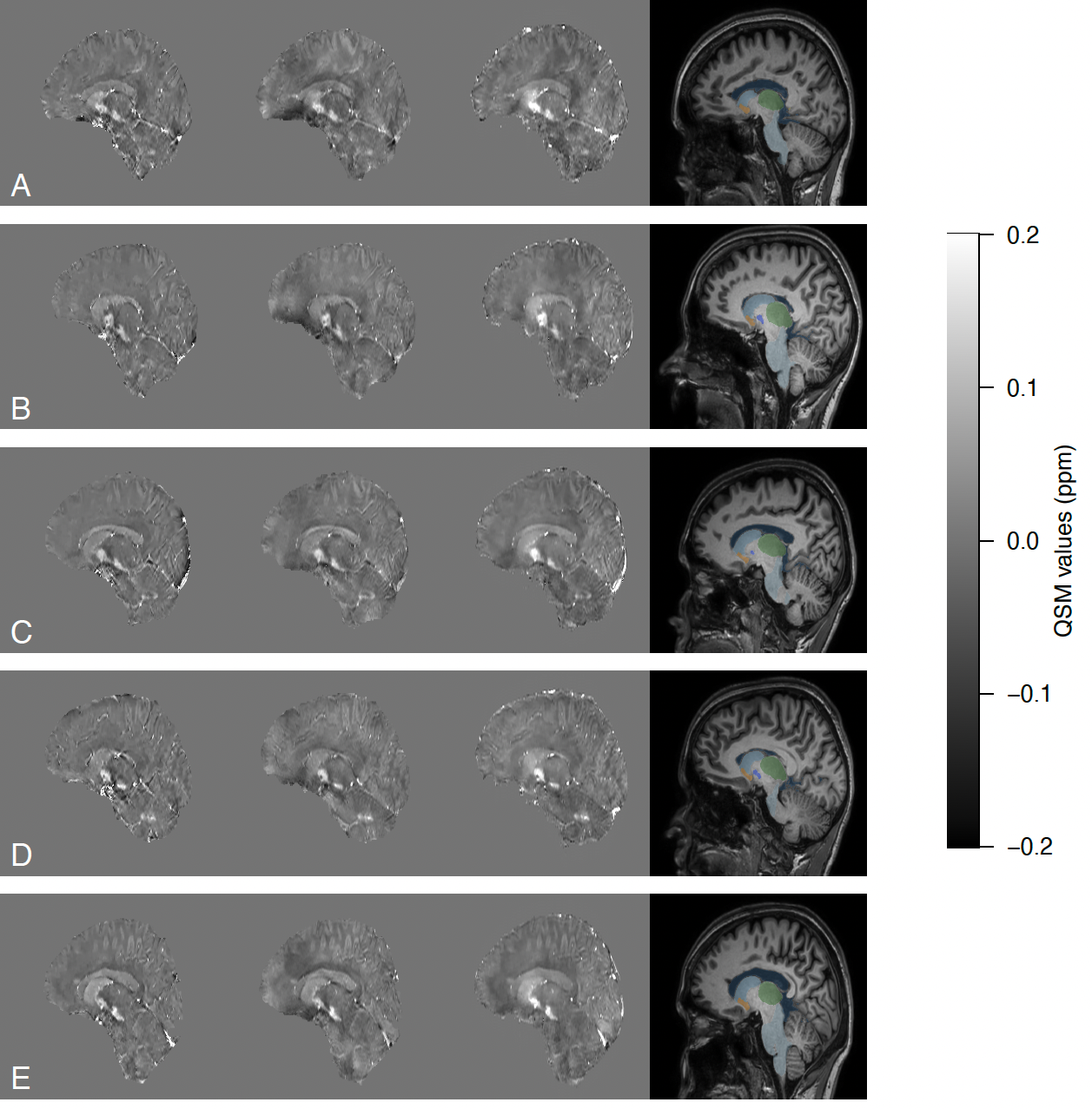


Sagittal plane comparison of QSM images

QSM images from three reconstruction pipelines (left to right: MEDI, STI-iLSQR, and mTFI) are compared. T1-weighted images are shown on the right for reference. Each row (A-E) displays images from one randomly selected patient.

**↑** Red arrows indicate streaking artifacts and bright artifacts from veins.

**↑** Yellow arrows indicate shadowing artifacts.

**↑** Orange arrows indicate differences in brain erosion of the superficial brain areas.

Abbreviations: DGM = deep grey matter; MEDI = Morphology Enabled Dipole Inversion; STI-iLSQR = STI-iterative least squares method; mTFI = multiecho Complex Total Field Inversion, T1w = T1-weighted.

## Supplementary Figure 3

| **MEDI** | **iLSQR** | **mTFI** | **T1w + DGM Mask** |
| --- | --- | --- | --- |


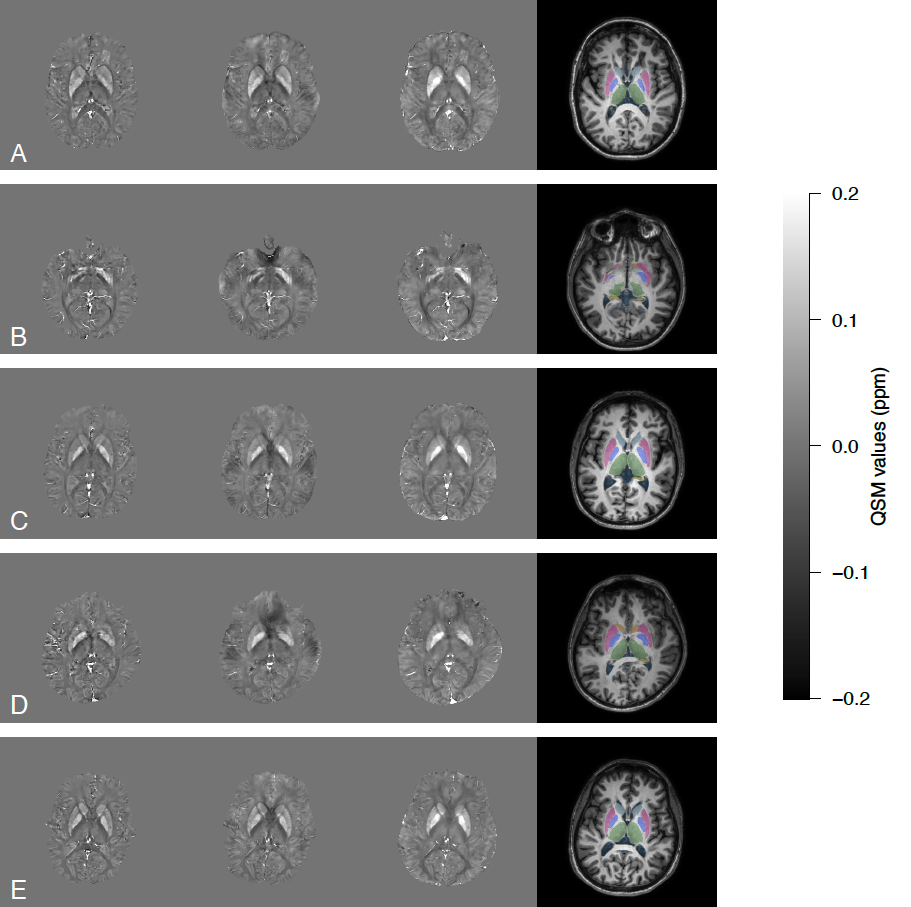


Horizontal plane comparison of QSM images

QSM images from three reconstruction pipelines (left to right: MEDI, STI-iLSQR, and mTFI) are compared. T1-weighted images are shown on the right for reference. Each row (A-E) displays images from one randomly selected patient.

**↑** Red arrows indicate streaking artifacts and bright artifacts from veins.

**↑** Yellow arrows indicate shadowing artifacts.

**↑** Orange arrows indicate differences in brain erosion of the superficial brain areas.

Abbreviations: DGM = deep grey matter; MEDI = Morphology Enabled Dipole Inversion; STI-iLSQR = STI-iterative least squares method; mTFI = multiecho Complex Total Field Inversion, T1w = T1-weighted.

## Supplementary Figure 4

| **Reason of Removal** | **QSM** | **Magnitude (First Echo)** |
| --- | --- | --- |
| Brain mask erosion | 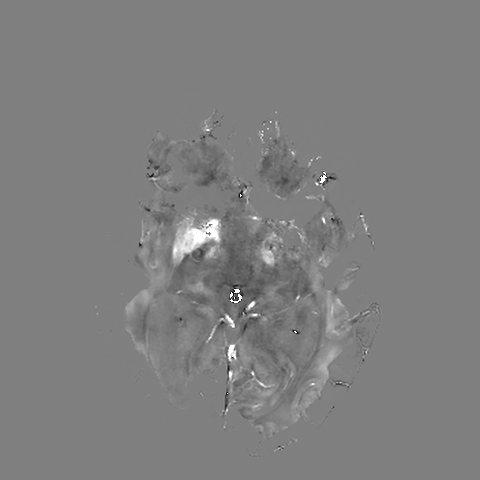 | 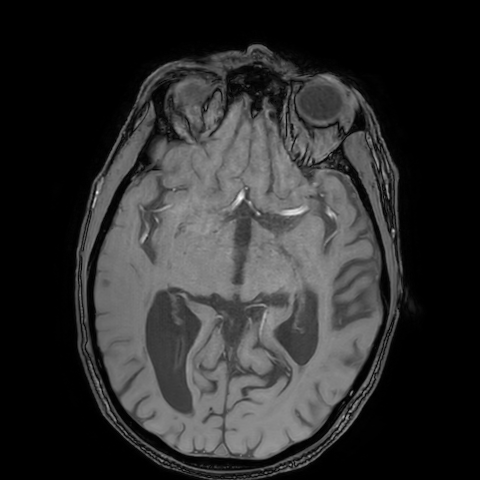 |
| Movement artifacts | 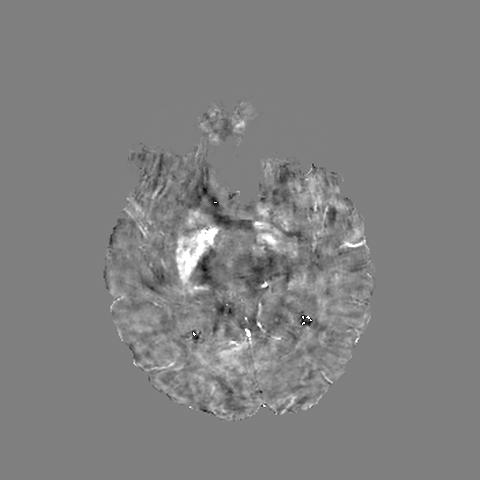 | 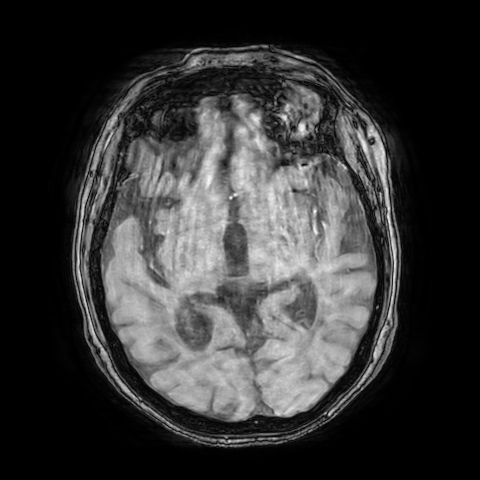 |
|  | 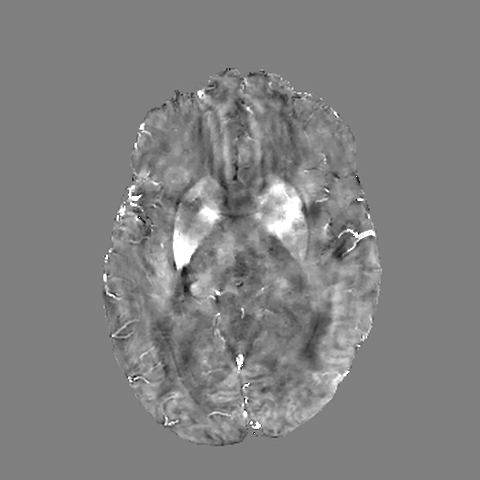 | 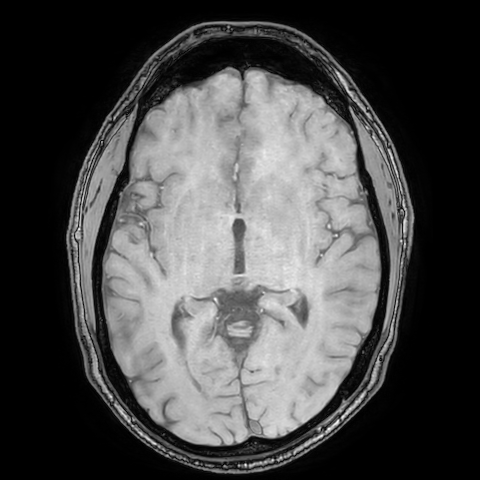 |
| Brain structure abnormalities | 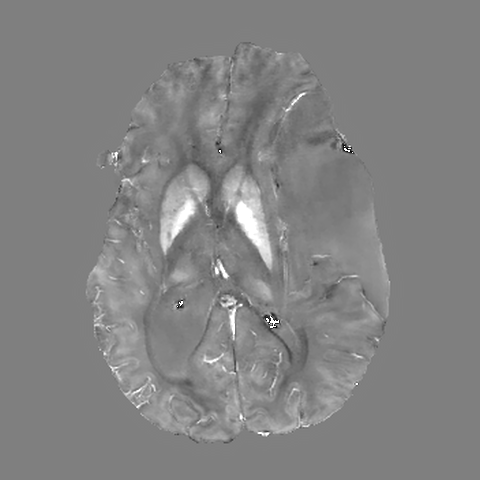 | 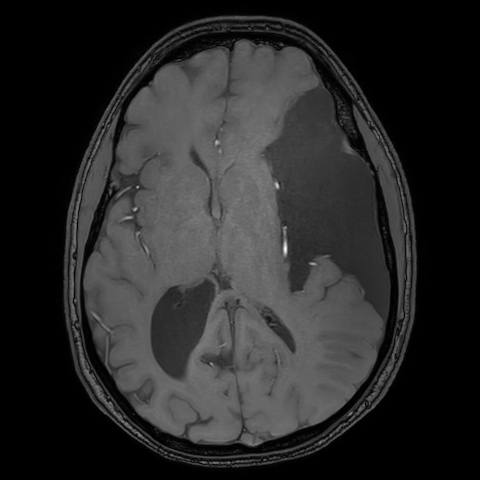 |

Examples of QSM images excluded from the study cohort.

QSM scans and their corresponding first echo magnitude images are presented, illustrating three conditions in which scans from MS patients were excluded due to artifacts or brain abnormalities (in this example, arachnoidal cyst).

# Section 4 Detailed Data Analysis and Reporting

## Supplementary Figure 5

Correlation Matrix for all baseline variables


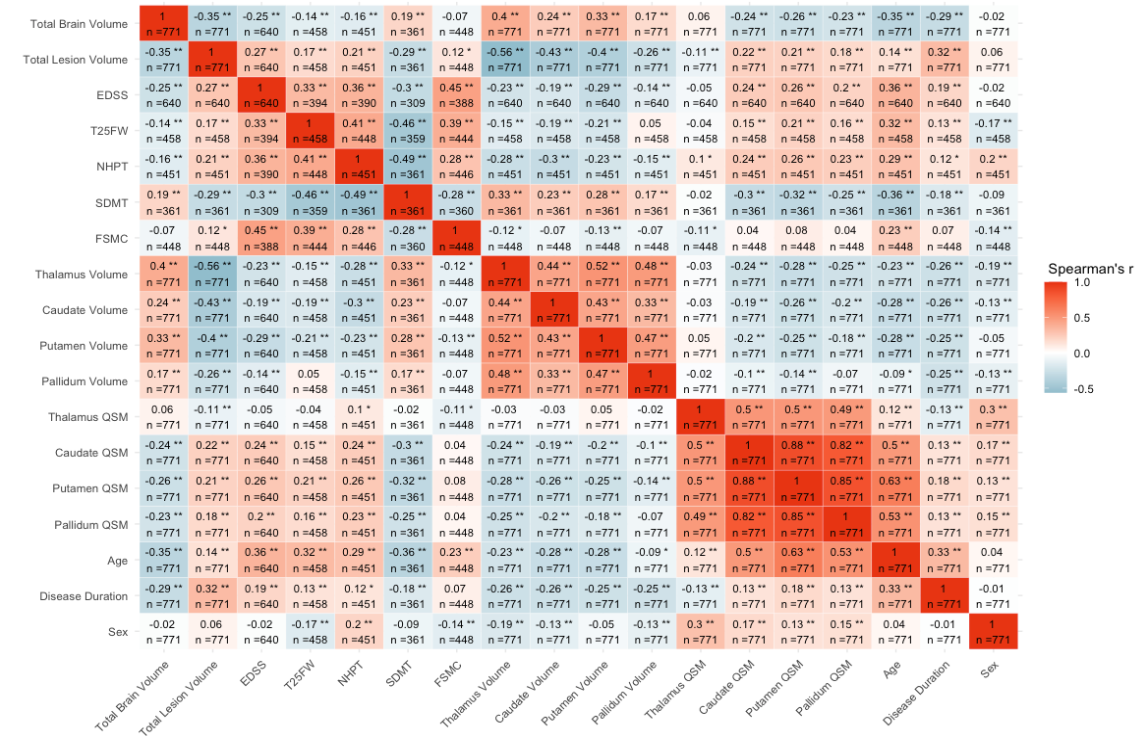


Supplementary Figure 4 shows simple correlations of all baseline variables, with false discovery rate corrections, * indicate p < 0.05, ** indicate p < 0.01

Total lesion volume was logarithmically transformed, sex was coded with female as 0 and male as 1.

Abbreviations: EDSS = Expanded Disability Status Scale, MSFC = Multiple Sclerosis Functional Composite, T25FW = Timed 25-Foot Walk, NHPT = Nine-Hole Peg Test, SDMT = Symbol Digit Modalities Test, FSMC = Fatigue Scale for Motor and Cognitive Functions.

## Supplementary Figure 6

Cross-sectional analyses – two separate regressional models linking **either** deep grey matter QSM values **or** regional volumes to clinical scores and MRI surrogates


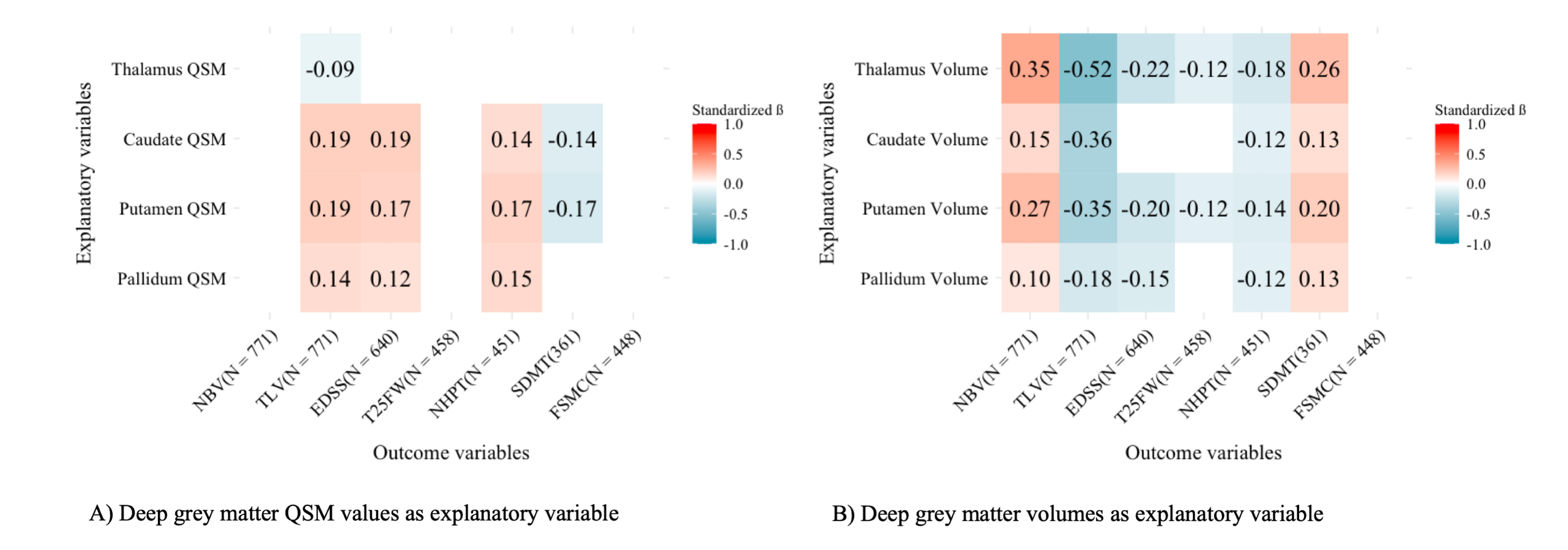


A) Regression model using only DGM QSM values as explanatory variable, modeled as: Outcome variable = β o + **β1 (QSM values)** + β2 (age) + β3 (sex) + β4 (disease duration) + ϵ .

B) Regression model using only regional DGM volumes as explanatory variable, modeled as: Outcome variable = β o **+ β1 (regional volume)** + β2 (age) + β3 (sex) + β4 (disease duration) + ϵ

The color grids represent standardized beta values from multiple linear regression models, indicating significant associations between DGM QSM values in (A) or regional DGM volumes (B) with clinical scores and MRI surrogates. All regression models were subjected to permutation testing and corrected for multiple testing using the false discovery rate correction.

Abbreviations: DGM = deep grey matter, EDSS = Expanded Disability Status Scale, FSMC = Fatigue Scale for Motor and Cognitive Functions, NBV = normalized brain volume, SDMT = Symbol Digit Modalities Test, Standardized β = Standardized beta coefficient, T25FW = Timed 25-Foot Walk, TLV = total lesion volume

Supplementary Figure 7

Cross-sectional analyses – one regression model linking deep grey matter QSM values **and** regional volumes with clinical scores and MRI surrogates


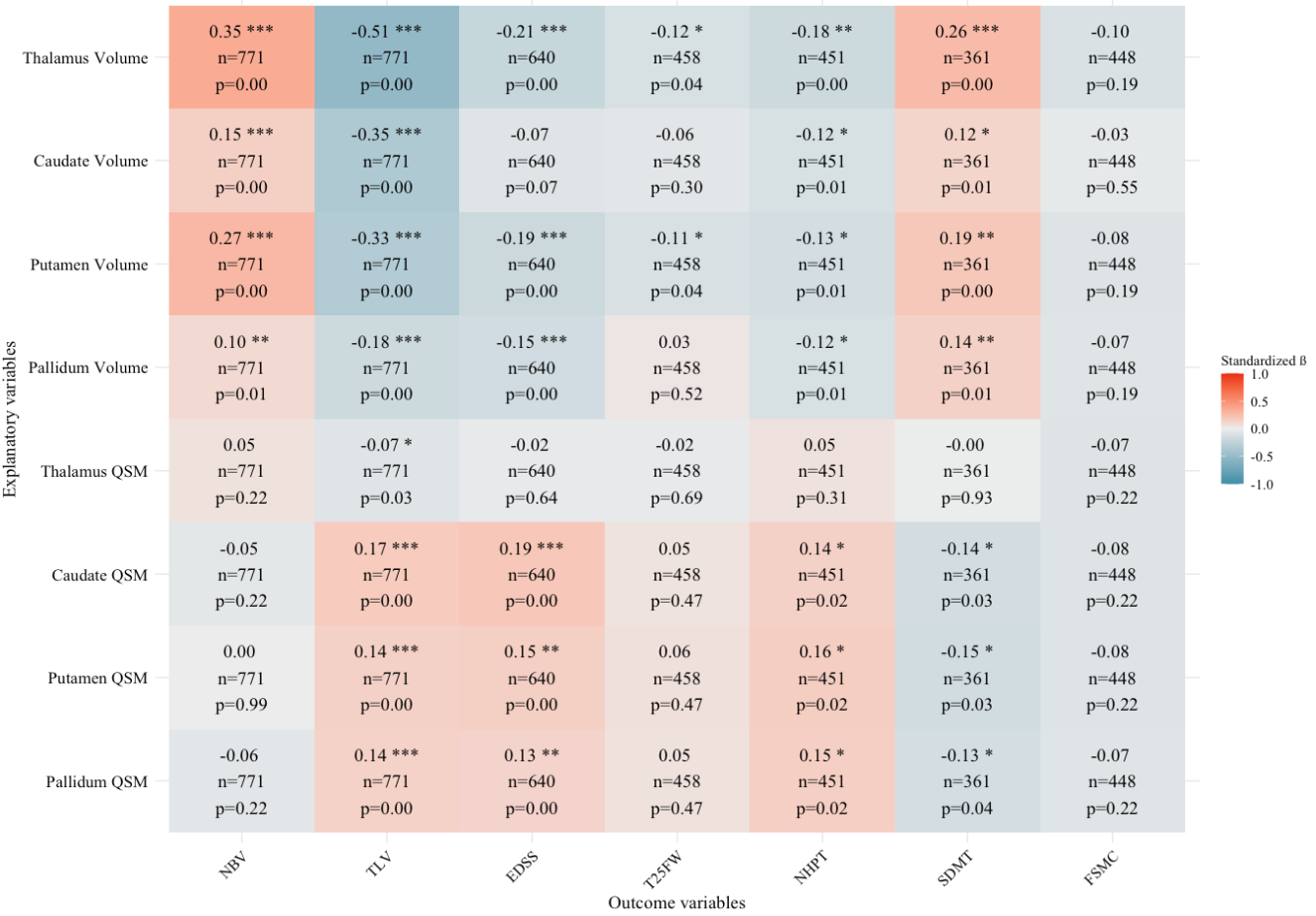


Color grids represent standardized beta of the cross-sectional associations of each regional deep grey matter (DGM) volume and QSM values from multiple linear regression models, with age, sex, and disease duration at baseline as additional covariates. All regression models were subjected to permutation testing and corrected for multiple testing using the false discovery rate correction.

* indicates p < 0.05; ** indicates p < 0.01, *** p < 0.001

Abbreviations: EDSS = Expanded Disability Status Scale, FSMC = Fatigue Scale for Motor and Cognitive Functions, NBV = normalized brain volume, SDMT = Symbol Digit Modalities Test, Standardized β = Standardized beta coefficient, T25FW = Timed 25-Foot Walk, TLV = total lesion volume.

Supplementary Figure 8

Cross-sectional regression model estimates

| Outcome Variables | DGM region |  |  | Regression models estimates | | | |
| --- | --- | --- | --- | --- | --- | --- | --- |
|  |  | R^2^ | Adjusted R^2^ | Range of Permuted Adjusted R^2^ | F-statistics |  | p-value |
| NBV | Thalamus | 0.275 | 0.270 | [-0.0065, 0.0274] | 57.99 | [0.0123, 5.3406] | 3.42E-51 |
|  | Caudate | 0.193 | 0.188 | [-0.0064, 0.0241] | 36.59 | [0.0135, 4.8003] | 1.17E-33 |
|  | Putamen | 0.236 | 0.231 | [-0.0065, 0.0268] | 47.35 | [0.0123, 5.2468] | 1.06E-42 |
|  | Pallidum | 0.182 | 0.177 | [-0.0064, 0.0239] | 34.03 | [0.0148, 4.7775] | 1.95E-31 |
| TLV | Thalamus | 0.351 | 0.346 | [-0.0065, 0.0281] | 82.59 | [0.0073, 5.456] | 2.31E-69 |
|  | Caudate | 0.259 | 0.254 | [-0.0064, 0.0252] | 53.38 | [0.0176, 4.9772] | 1.44E-47 |
|  | Putamen | 0.240 | 0.235 | [-0.0065, 0.0268] | 48.24 | [0.0055, 5.2387] | 2.00E-43 |
|  | Pallidum | 0.167 | 0.162 | [-0.0064, 0.0322] | 30.71 | [0.0177, 6.1306] | 1.60E-28 |
| EDSS | Thalamus | 0.178 | 0.171 | [-0.0077, 0.0386] | 27.41 | [0.018, 6.1311] | 3.75E-25 |
|  | Caudate | 0.172 | 0.166 | [-0.0078, 0.033] | 26.41 | [0.0111, 5.3678] | 2.79E-24 |
|  | Putamen | 0.189 | 0.182 | [-0.0078, 0.0348] | 29.48 | [0.0106, 5.6133] | 5.95E-27 |
|  | Pallidum | 0.173 | 0.167 | [-0.0077, 0.0321] | 26.60 | [0.0173, 5.233] | 1.92E-24 |
| T25FW | Thalamus | 0.140 | 0.130 | [-0.011, 0.0496] | 14.71 | [0.0083, 5.7693] | 2.26E-13 |
|  | Caudate | 0.133 | 0.124 | [-0.011, 0.0723] | 13.88 | [0.0077, 8.1276] | 1.24E-12 |
|  | Putamen | 0.142 | 0.133 | [-0.0109, 0.0457] | 14.97 | [0.0166, 5.3811] | 1.31E-13 |
|  | Pallidum | 0.132 | 0.122 | [-0.0109, 0.0496] | 13.69 | [0.0132, 5.7666] | 1.85E-12 |
| NHPT | Thalamus | 0.096 | 0.086 | [-0.0111, 0.0425] | 9.48 | [0.0119, 4.9992] | 1.34E-08 |
|  | Caudate | 0.098 | 0.088 | [-0.0109, 0.053] | 9.70 | [0.0266, 6.0331] | 8.32E-09 |
|  | Putamen | 0.102 | 0.092 | [-0.011, 0.0559] | 10.07 | [0.02, 6.3253] | 3.77E-09 |
|  | Pallidum | 0.101 | 0.091 | [-0.0111, 0.0594] | 9.96 | [0.0134, 6.6868] | 4.81E-09 |
| SDMT | Thalamus | 0.205 | 0.193 | [-0.0139, 0.0579] | 18.26 | [0.0143, 5.4215] | 3.92E-16 |
|  | Caudate | 0.185 | 0.174 | [-0.0138, 0.0605] | 16.14 | [0.0166, 5.6376] | 2.43E-14 |
|  | Putamen | 0.201 | 0.190 | [-0.0139, 0.0514] | 17.84 | [0.01, 4.9018] | 8.76E-16 |
|  | Pallidum | 0.186 | 0.174 | [-0.0138, 0.0574] | 16.22 | [0.0166, 5.3858] | 2.07E-14 |
| FSMC | Thalamus | 0.098 | 0.088 | [-0.0112, 0.0433] | 9.61 | [0.006, 5.0495] | 1.02E-08 |
|  | Caudate | 0.091 | 0.080 | [-0.0112, 0.0475] | 8.80 | [0.0087, 5.4547] | 5.69E-08 |
|  | Putamen | 0.093 | 0.083 | [-0.0109, 0.0568] | 9.10 | [0.0357, 6.3857] | 2.98E-08 |
|  | Pallidum | 0.094 | 0.083 | [-0.0112, 0.0462] | 9.12 | [0.0111, 5.3349] | 2.87E-08 |

Abbreviations: EDSS = Expanded Disability Status Scale, FSMC = Fatigue Scale for Motor and Cognitive Functions, NBV = normalized brain volume, SDMT = Symbol Digit Modalities Test, , T25FW = Timed 25-Foot Walk, TLV = total lesion volume

## Supplementary Figure 9

Longitudinal analyses - two separate regression models linking **either** baseline deep grey matter QSM values **or** baseline regional volumes to follow-up clinical scores and MRI surrogates


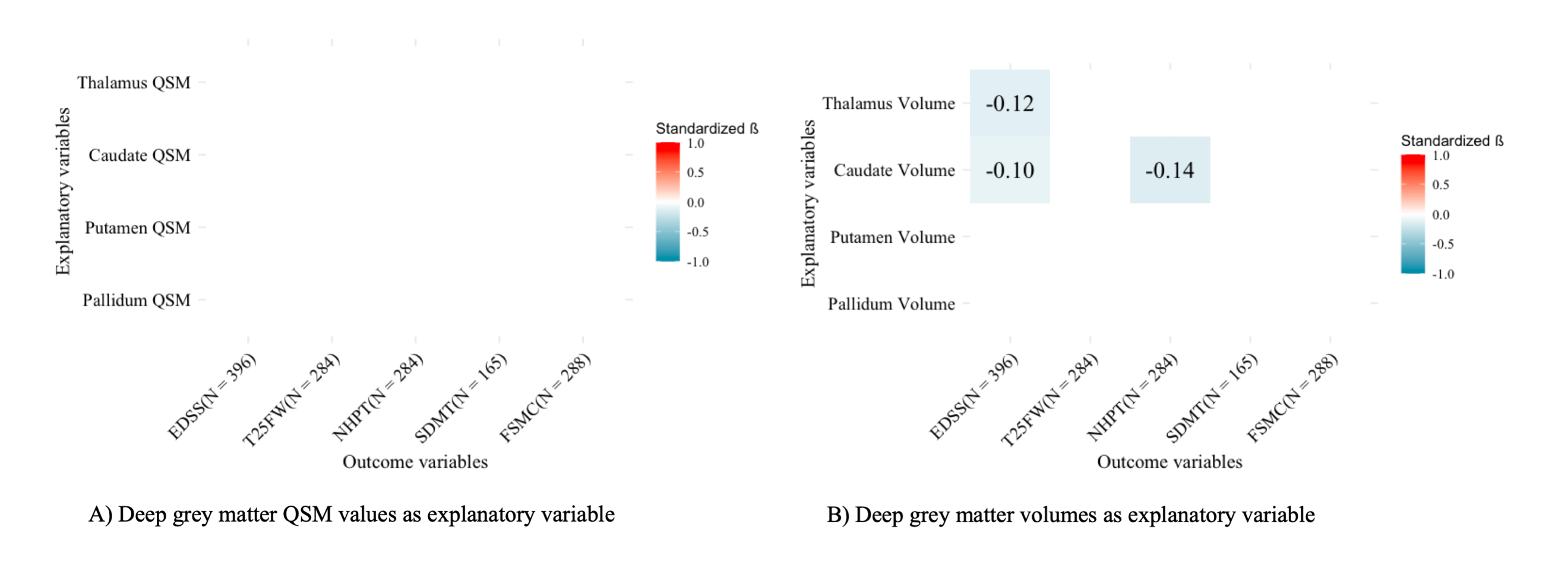


A) Regression model using only DGM QSM values as explanatory variable, modeled as: Outcome variable at follow-up = βo + **β1 (baseline QSM values)** + β2 (baseline age) + β3 (sex) + β4 (baseline disease duration) + β5 (baseline outcome variable) + β6 (follow-up interval) + ϵ .

B) Regression model using only regional DGM volumes as explanatory variable, modeled as: Outcome variable at follow-up = βo **+ β1 (baseline regional volume)** + β2 (baseline age) + β3 (sex) + β4 (baseline disease duration) + β5 (baseline outcome variable) + β6 (follow-up interval) + ϵ

The color grids represent standardized beta values from multiple linear regression models, indicating significant associations between baseline DGM QSM values in (A) or baseline regional volumes (B) with follow-up clinical scores and MRI surrogates. All regression models were subjected to permutation testing and corrected for multiple testing using the false discovery rate correction.

Color grids represent standardized beta of the cross-sectional associations of each regional deep grey matter (DGM) volume and QSM values from multiple linear regression models, with age, sex, and disease duration at baseline as additional covariates.

All regression models were subjected to permutation testing and corrected for multiple testing using the false discovery rate correction.

* indicates p < 0.05; ** indicates p < 0.01, *** p < 0.001

Abbreviations: EDSS = Expanded Disability Status Scale, FSMC = Fatigue Scale for Motor and Cognitive Functions, NBV = normalized brain volume, SDMT = Symbol Digit Modalities Test, Standardized β = Standardized beta coefficient, T25FW = Timed 25-Foot Walk, TLV = total lesion volume.

## Supplementary Figure 10

Longitudinal analyses – one regression model linking baseline deep grey matter QSM values **and** regional volumes with follow-up clinical scores


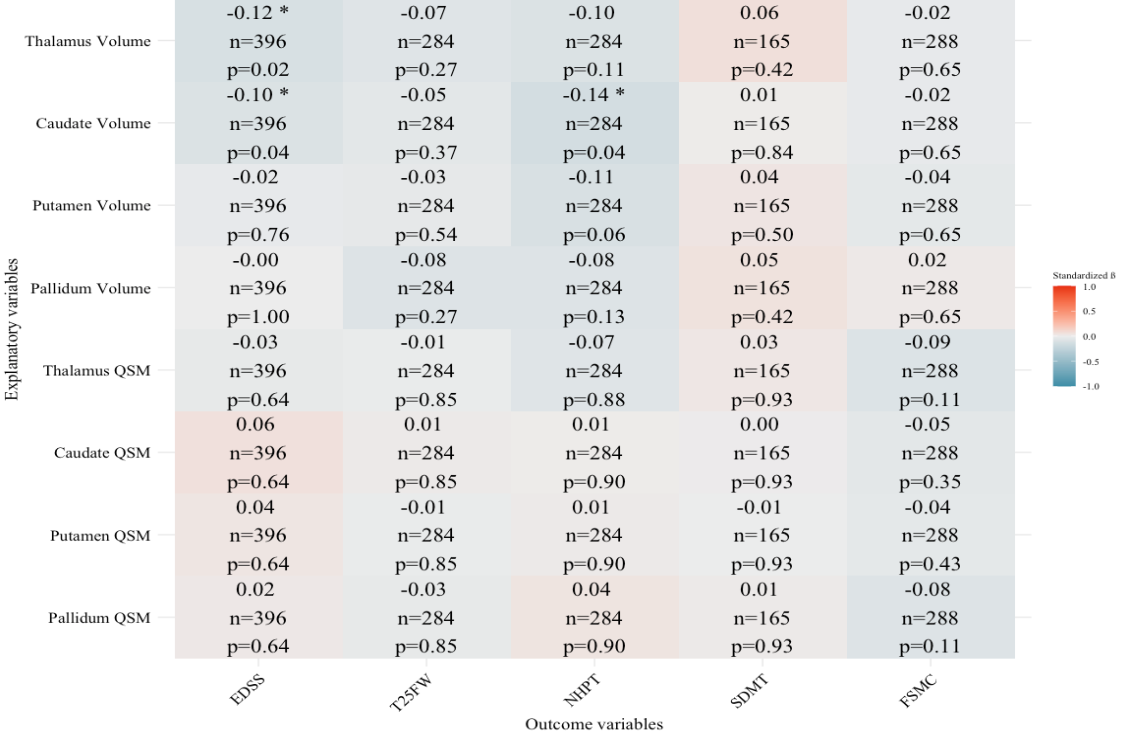


Color grids represent the standardized beta values of longitudinal regression models after permutation testing and false discovery rate corrections.

* indicates p < 0.05; ** indicates p < 0.01, *** p < 0.001

Each column represents associations of one outcome variable at the follow-up time point, with four different regional deep grey matter (DGM) volumes and QSM values, confounding variables such as age and sex were adjusted. All longitudinal regression models were also corrected for their respective baseline clinical scores as well as follow-up durations.

Abbreviations: EDSS = Expanded Disability Status Scale, FSMC = Fatigue Scale for Motor and Cognitive Functions, SDMT = Symbol Digit Modalities Test, Standardized β = Standardized beta coefficient, T25FW = Timed 25-Foot Walk, NHPT = Nine-Hole Peg Test

Supplementary Figure 11

Longitudinal regression model estimates

| Outcome Variables | DGM region |  |  | | Regression models estimates | | | |
| --- | --- | --- | --- | --- | --- | --- | --- | --- |
|  |  | R^2^ | Adjusted R^2^ | Range of Permuted Adjusted R^2^ | | F-statistics | Range of Permuted F-statistics | p-value  (F-statistics) |
| EDSS | Thalamus | 0.429 | 0.419 | [-0.0222 - 0.0758] | | 41.61 | [0.041 - 4.583] | 1.30E-43 |
|  | Caudate | 0.427 | 0.417 | [-0.0225 - 0.0831] | | 41.28 | [0.038 - 4.973] | 2.48E-43 |
|  | Putamen | 0.418 | 0.407 | [-0.0225 - 0.0638] | | 39.74 | [0.022 - 4.016] | 5.30E-42 |
|  | Pallidum | 0.417 | 0.406 | [-0.0221 - 0.0926] | | 39.58 | [0.044 - 5.606] | 7.29E-42 |
| T25FW | Thalamus | 0.477 | 0.464 | [-0.0447 - 0.1596] | | 35.94 | [0.052 - 5.313] | 1.63E-35 |
|  | Caudate | 0.475 | 0.462 | [-0.0471 - 0.1231] | | 35.66 | [0.05 - 4.069] | 2.73E-35 |
|  | Putamen | 0.473 | 0.460 | [-0.0442 - 0.1336] | | 35.43 | [0.043 - 4.467] | 4.14E-35 |
|  | Pallidum | 0.479 | 0.465 | [-0.0457 - 0.1391] | | 36.19 | [0.032 - 4.554] | 1.06E-35 |
| NHPT | Thalamus | 0.348 | 0.332 | [-0.0458 - 0.2471] | | 21.05 | [0.018 - 8.362] | 1.19E-22 |
|  | Caudate | 0.354 | 0.337 | [-0.0452 - 0.2208] | | 21.56 | [0.056 - 7.233] | 3.86E-23 |
|  | Putamen | 0.348 | 0.331 | [-0.0456 - 0.2265] | | 21.04 | [0.054 - 7.694] | 1.21E-22 |
|  | Pallidum | 0.343 | 0.327 | [-0.0451 - 0.214] | | 20.60 | [0.042 - 7.033] | 3.23E-22 |
| SDMT | Thalamus | 0.759 | 0.748 | [-0.1299 - 0.3741] | | 70.45 | [0.042 - 5.098] | 3.17E-45 |
|  | Caudate | 0.755 | 0.744 | [-0.1364 - 0.4062] | | 68.93 | [0.03 - 6.571] | 1.14E-44 |
|  | Putamen | 0.756 | 0.745 | [-0.153 - 0.4031] | | 69.39 | [0.039 - 6.209] | 7.73E-45 |
|  | Pallidum | 0.757 | 0.746 | [-0.1386 - 0.3052] | | 69.93 | [0.039 - 4.749] | 4.88E-45 |
| FSMC | Thalamus | 0.642 | 0.633 | [-0.0418 - 0.1111] | | 71.76 | [0.042 - 4.175] | 8.12E-59 |
|  | Caudate | 0.638 | 0.629 | [-0.0422 - 0.1159] | | 70.43 | [0.022 - 4.09] | 4.25E-58 |
|  | Putamen | 0.637 | 0.628 | [-0.0399 - 0.1216] | | 70.32 | [0.064 - 4.381] | 4.88E-58 |
|  | Pallidum | 0.641 | 0.632 | [-0.0405 - 0.1329] | | 71.30 | [0.037 - 4.896] | 1.44E-58 |

Abbreviations: EDSS = Expanded Disability Status Scale, FSMC = Fatigue Scale for Motor and Cognitive Functions, SDMT = Symbol Digit Modalities Test, Standardized β = Standardized beta coefficient, T25FW = Timed 25-Foot Walk

# References

1. Boehm C, Sollmann N, Meineke J, et al (2022) Preconditioned water‐fat total field inversion: Application to spine quantitative susceptibility mapping. Magnetic Resonance in Med 87:417–430. https://doi.org/10.1002/mrm.28903

2. Wen Y, Spincemaille P, Nguyen T, et al (2021) Multiecho complex total field inversion method (mcTFI) for improved signal modeling in quantitative susceptibility mapping. Magnetic Resonance in Med 86:2165–2178. https://doi.org/10.1002/mrm.28814

3. Liu T, Xu WY, Spincemaille P, et al (2012) Accuracy of the Morphology Enabled Dipole Inversion (MEDI) Algorithm for Quantitative Susceptibility Mapping in MRI. IEEE Trans Med Imaging 31:816–824. https://doi.org/10.1109/TMI.2011.2182523

4. Schweser F, Robinson SD, De Rochefort L, et al (2017) An illustrated comparison of processing methods for phase MRI and QSM: removal of background field contributions from sources outside the region of interest. NMR in Biomedicine 30:e3604. https://doi.org/10.1002/nbm.3604

5. Liu Z, Kee Y, Zhou D, et al (2017) Preconditioned total field inversion (TFI) method for quantitative susceptibility mapping. Magnetic Resonance in Med 78:303–315. https://doi.org/10.1002/mrm.26331

6. Geerts-Ossevoort L, de Weerdt E, Duijndam A, et al (2020) Speed done right. Every time.

7. Wang Z, Mak HK, Cao P (2023) Deep learning‐regularized, single‐step quantitative susceptibility mapping quantification. NMR in Biomedicine 36:. https://doi.org/10.1002/nbm.4849

8. Berg RC, Preibisch C, Thomas DL, et al (2021) Investigating the effect of flow compensation and quantitative susceptibility mapping method on the accuracy of venous susceptibility measurement. NeuroImage 240:118399. https://doi.org/10.1016/j.neuroimage.2021.118399
